# Leveraging a physiologically-based quantitative translational modeling platform for designing B cell maturation antigen-targeting bispecific T cell engagers for treatment of multiple myeloma

**DOI:** 10.1371/journal.pcbi.1009715

**Published:** 2022-07-15

**Authors:** Tomoki Yoneyama, Mi-Sook Kim, Konstantin Piatkov, Haiqing Wang, Andy Z. X. Zhu

**Affiliations:** 1 Quantitative Solutions, Takeda Pharmaceuticals International Co., Cambridge, Massachusetts, United States of America; 2 Global Drug Metabolism and Pharmacokinetics, Takeda Pharmaceuticals International Co., Cambridge, Massachusetts, United States of America; Tel Aviv University, UNITED STATES

## Abstract

Bispecific T cell engagers (TCEs) are an emerging anti-cancer modality that redirects cytotoxic T cells to tumor cells expressing tumor-associated antigens (TAAs), thereby forming immune synapses to exert anti-tumor effects. Designing pharmacokinetically acceptable TCEs and optimizing their size presents a considerable protein engineering challenge, particularly given the complexity of intercellular bridging between T cells and tumor cells. Therefore, a physiologically-relevant and clinically-verified computational modeling framework is of crucial importance to understand the protein engineering trade-offs. In this study, we developed a quantitative, physiologically-based computational framework to predict immune synapse formation for a variety of molecular formats of TCEs in tumor tissues. Our model incorporates a molecular size-dependent biodistribution using the two-pore theory, extravasation of T cells and hematologic cancer cells, mechanistic bispecific intercellular binding of TCEs, and competitive inhibitory interactions by shed targets. The biodistribution of TCEs was verified by positron emission tomography imaging of [^89^Zr]AMG211 (a carcinoembryonic antigen-targeting TCE) in patients. Parameter sensitivity analyses indicated that immune synapse formation was highly sensitive to TAA expression, degree of target shedding, and binding selectivity to tumor cell surface TAAs over shed targets. Notably, the model suggested a “sweet spot” for TCEs’ CD3 binding affinity, which balanced the trapping of TCEs in T-cell-rich organs. The final model simulations indicated that the number of immune synapses is similar (~55/tumor cell) between two distinct clinical stage B cell maturation antigen (BCMA)-targeting TCEs, PF-06863135 in an IgG format and AMG420 in a BiTE format, at their respective efficacious doses in multiple myeloma patients. This result demonstrates the applicability of the developed computational modeling framework to molecular design optimization and clinical benchmarking for TCEs, thus suggesting that this framework can be applied to other targets to provide a quantitative means to facilitate model-informed best-in-class TCE discovery and development.

## Author summary

Cytotoxic T cells play a crucial role in eliminating tumor cells. However, tumor cells develop mechanisms to evade T cell recognition. Bispecific T cell engagers (TCEs) are designed to overcome this issue by bringing T cells into close proximity of tumor cells through simultaneous bispecific binding to both tumor-associated antigens and T cells. After successful regulatory approval of catumaxomab (an anti-EpCAM TCE, withdrawn in 2017) and blinatumomab (an anti-CD19 TCE), more than 40 TCEs are currently in clinical development with a variety of molecular sizes and protein formats. In this study, we developed a quantitative computational modeling framework for molecular design optimization and clinical benchmarking of TCEs. The model accounts for molecular size-dependent biodistribution of TCEs to tumor tissues and other organs as well as subsequent bispecific intercellular bridging of T cells and tumor cells. The model simulation highlighted the importance of binding selectivity of TCEs to tumor cell surface targets over shed targets. The model also demonstrated a good agreement in predicted immune synapse number for two distinct molecular formats of TCEs at their respective clinically efficacious dose levels, thus highlighting the usefulness of a developed computational modeling framework for best-in-class TCE discovery and development.

## 1 Introduction

The past decade revealed the remarkable potential of cancer immunotherapies, including immune checkpoint inhibitors [1,2], chimeric antigen receptor engineered T cells (CAR-T) [3], oncolytic viruses [4], and bispecific T cell engagers (TCEs) [5–10]. TCEs are an emerging anti-cancer modality that redirects cytotoxic T cells to tumor cells expressing tumor-associated antigens (TAAs), thereby forming immune synapses to activate and cause proliferation of T cells near the tumor cells. The activated T cells then exert anti-tumor effects through release of perforin for membrane perforation followed by granzyme-mediated apoptotic death of the engaged tumor cells. An important advantage of TCEs over other immunotherapies is that they directly activate T cells through cluster of differentiation 3 (CD3) signaling, thus making it possible to bypass major histocompatibility complex (MHC) restrictions and antigen specificity of the T cell receptors (TCR). After successful regulatory approval of catumaxomab (an anti-epithelial cell adhesion molecule [EpCAM] TCE, withdrawn in 2017) and blinatumomab (an anti-CD19 TCE), more than 40 TCEs are currently in clinical development and over 100 others are under preclinical development targeting both hematologic malignancies and solid tumors [7].

Due to the complex bispecific mechanism of action of TCEs as well as a lack of target cross-reactivity between preclinical animal models, typically mice, and humans for efficacy, computational models provide a crucial means to better understand the efficacy impact of drug-related parameters such as binding affinities to TAAs/CD3s and protein formats as well as physiological expression levels of TAAs/CD3s in target tissues of interest. A number of quantitative computational modeling frameworks have been developed and published within the last few years to quantitatively and translationally address these challenges. The earliest attempts started with modeling of in vitro experimental results of TCE-induced cytotoxicity of tumor cells and simultaneous T cell activation. The observed data were successfully delineated by a mathematical modeling framework that accounted for bispecific binding to cell surface TAAs on tumor cells and CD3s on T cells under a wide range of TCE concentrations as well as variable effector-to-tumor-cell (E:T) ratios [11]. Jiang and colleagues further demonstrated that in vitro cytotoxicity results are predictive of clinical efficacy of blinatumomab by considering the difference in concentrations of tumor cells and T cells between in vitro experimental conditions and patients in vivo [12]. Subsequently, modeling frameworks were developed to characterize in vivo responses of TCEs in the monkey as well as the human peripheral blood mononuclear cells (PBMC) transferred mouse model [13–15]. Recently, a more integrated, clinically-oriented modeling framework has been published that incorporates biodistribution of TCEs as well as tissue expression gradient of target tumor cells and T cells [16,17] and further connects with a large cancer-immunity cycle model that is verified to immune checkpoint inhibitors [18].

However, most of the modeling frameworks published thus far are not designed to compare different molecular sizes and protein formats of TCEs, and there is a lack of quantitative verification of tissue distribution of TCEs under variable abundance of T cells in different tissues in patients. Owing to the advance in therapeutic protein engineering, next-generation TCE candidates comprise a variety of molecular sizes and structural domains. As a result, significant differences are expected in drug dispositions and biodistribution of those TCEs. In addition to a huge difference expected in elimination half-lives (a typical blood elimination half-life is 2–3 hours for blinatumomab, which uses a bispecific T cell engager [BiTE] format [fusion proteins consisting of two single-chain variable fragments] at molecular weight of 54 kDa [19], while it is 2–3 weeks for a full-length antibody at 150 kDa), biodistribution of therapeutic proteins is also reported to be influenced by molecular sizes [20]. Furthermore, although the influence of shed targets on pharmacokinetics (PK)/pharmacodynamics (PD) of targeting biologics has been well acknowledged [14], biodistribution of shed targets and their impact on the PK/PD relationship of TCEs at a site of action has not been fully characterized.

In light of the encouraging translational predictability of physiologically-based pharmacokinetic (PBPK) models for biodistribution of protein therapeutics, especially for monoclonal antibodies (mAbs) [21–23], extravasation models based on the two-pore theory have been proposed for biodistribution of different molecular sizes of proteins to account for more detailed physiological tissue distribution processes [24–26]. The two-pore theory assumes that the tissue vascular endothelium is “porous” and the pore radius on endothelial cells can be classified into two groups, namely a large amount of small pores (~4 nm) and a smaller number of large pores (~22 nm) with fractional hydraulic conductance at 0.958 and 0.042, respectively [21,27]. The biodistribution of proteins can be projected by explicitly estimating convectional and diffusional distribution rate constants between the vascular space and interstitial space of tissues through either small or large pores considering presence of a circular isogravimetric flow driven by the osmotic pressure. The mathematical modeling framework has been developed and verified by experimental data from studies in animals and humans to characterize biodistribution to a variety of tissues in a molecular size-dependent manner [24–26].

In this study, we have developed a quantitative, physiologically-based computational modeling framework for molecular designing of TCEs for a variety of molecular sizes and protein formats. Our platform model can characterize 1) molecular size-dependent biodistribution of TCEs and shed targets in blood, bone marrow, spleen, and lymph node under the two-pore biodistribution theory, 2) physiologically-based extravasation and biodistribution of T cells and tumor cells, and 3) mechanistic bispecific binding of TCEs to TAAs and T cells while accounting for competitive inhibitory binding to shed targets. The two-pore theory biodistribution model was translationally scaled up from mice to patients, and biodistribution of TCEs to bone marrow and spleen in patients was verified by clinical positron emission tomography (PET) imaging of [^89^Zr]AMG211, an anti-carcinoembryonic antigen (CEA)-targeting TCE in a BiTE format [28]. Additional model characterization was performed after calibrating the model for multiple myeloma and B cell maturation antigen (BCMA) as a phenotypical disease and target, respectively, to demonstrate the value of this platform. This disease/target combination was selected due to the large amount of literature data on different protein formats of anti-BCMA TCEs under clinical development (e.g., PF-06863135 in an IgG format and AMG420 in a BiTE format) as well as the abundance of information on shed BCMAs in circulating blood and its inhibitory effect against TCE-induced cytotoxicity [29]. The model was calibrated for selective binding property between tumor cell surface BCMAs and shed BCMAs estimated from in vitro cytotoxicity assays of both PF-06863135 and AMG420 in the presence of shed BCMAs based on data from published literature [29,30]. The calibrated platform was interrogated for key drug-related and system-related parameters for molecular design optimization of TCEs. Moreover, the platform model was applied to simulate and compare amounts of immune synapses between T cells and tumor cells in bone marrow, the tumor site of multiple myeloma patients, after treatment of distinct TCEs, PF-06863135 and AMG420, at their respective clinical efficacious dose levels to understand the amount of target engagement required in the clinic to treat multiple myeloma.

## 2 Results

### 2.1 Model-based characterization of in vitro cytotoxicity of TCEs in the presence of shed targets

**Fig 1:**
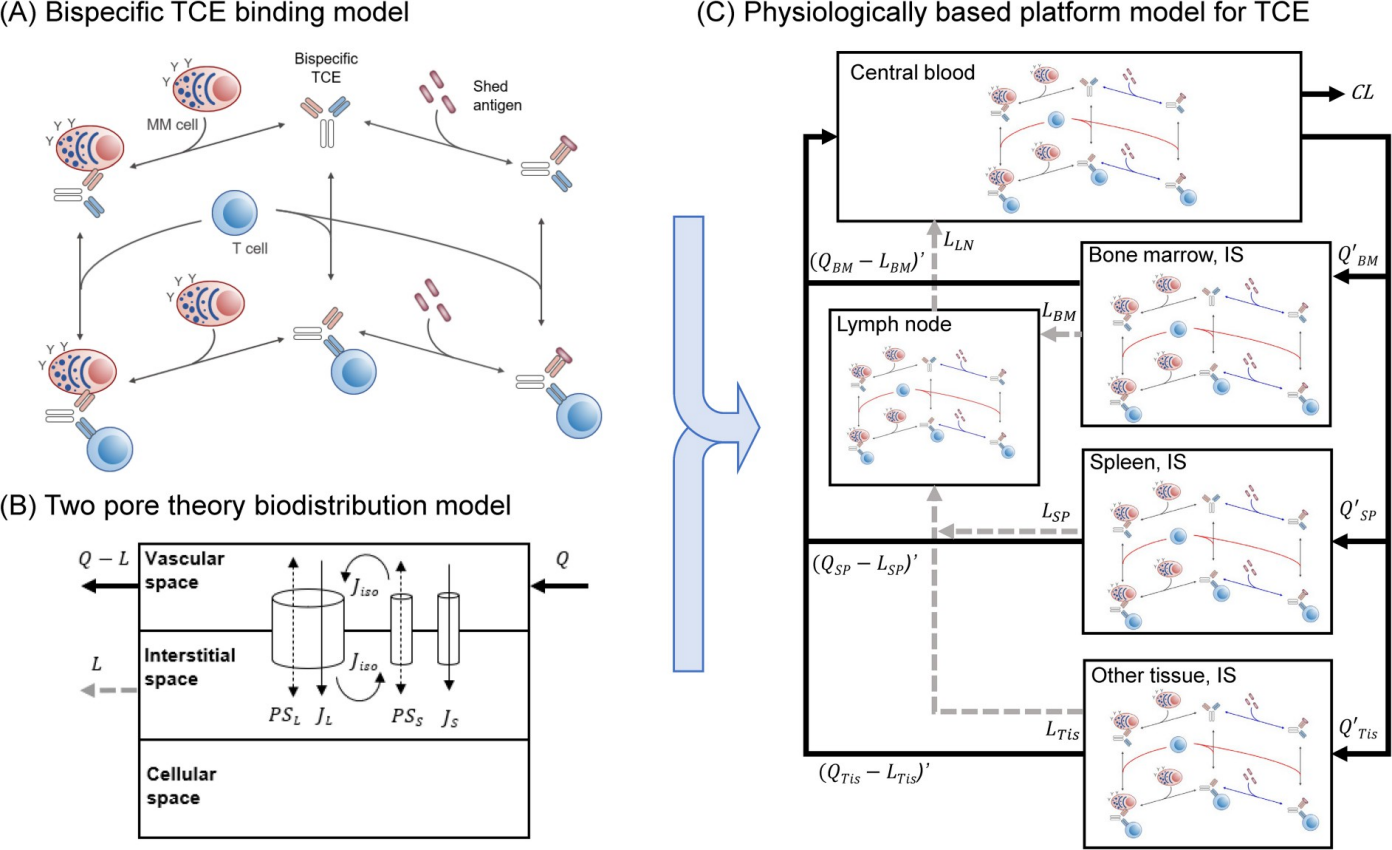
Schematic computational platform model structures for TCEs. (A) Mechanistic bispecific binding model of TCEs where bispecific TCEs bind either to tumor-associated antigens (TAAs) on tumor cells or to CD3s on T cells to form TCE-TAA or TCE-CD3 dimers, subsequently binding with other binding targets, either CD3s or TAAs, respectively, to form immune synapses (TAA-TCE-CD3 trimer). Shed targets competitively inhibit TCEs or TCE-CD3s binding to TAAs. (B) Two-pore theory biodistribution model. TCEs and shed targets are transported from vascular space to tissue interstitial space via two groups of pores (small pores: ~4.44 nm, large pores: ~22.9 nm) by diffusion and convection in a molecular size-dependent manner. Q, Q-L, and L represent an arterial blood flow, a venous blood flow, and a lymphatic flow in each tissue, respectively. PS, J, and J_iso_ represent diffusion, convection, and insogravimetric flows, respectively. Subscript L and S represent large and small pores. (C) Integrated physiologically-based platform model for TCEs. The biodistribution processes of TCEs and shed targets into and out of each tissue were characterized by integrated rate constants accounting for perfusion, diffusion, convection, and lymphatic flow rate constants in a molecular size-dependent manner based on the two-pore theory. Extravasation and biodistribution of T cells and multiple myeloma cells are incorporated into the model. In each tissue and blood compartment, bispecific TCE binding interactions between TAAs, CD3s, and shed targets were considered. Q’ and (Q-L)’ represent integrated biodistribution rate constants in and out of tissue interstitial spaces, respectively. L and CL represent lymphatic flows and a systemic clearance of TCEs or shed targets, respectively. Subscript BM, SP, LN, and Tis represent bone marrow, spleen, lymph node, and other tissue, respectively.

**Fig 2:**
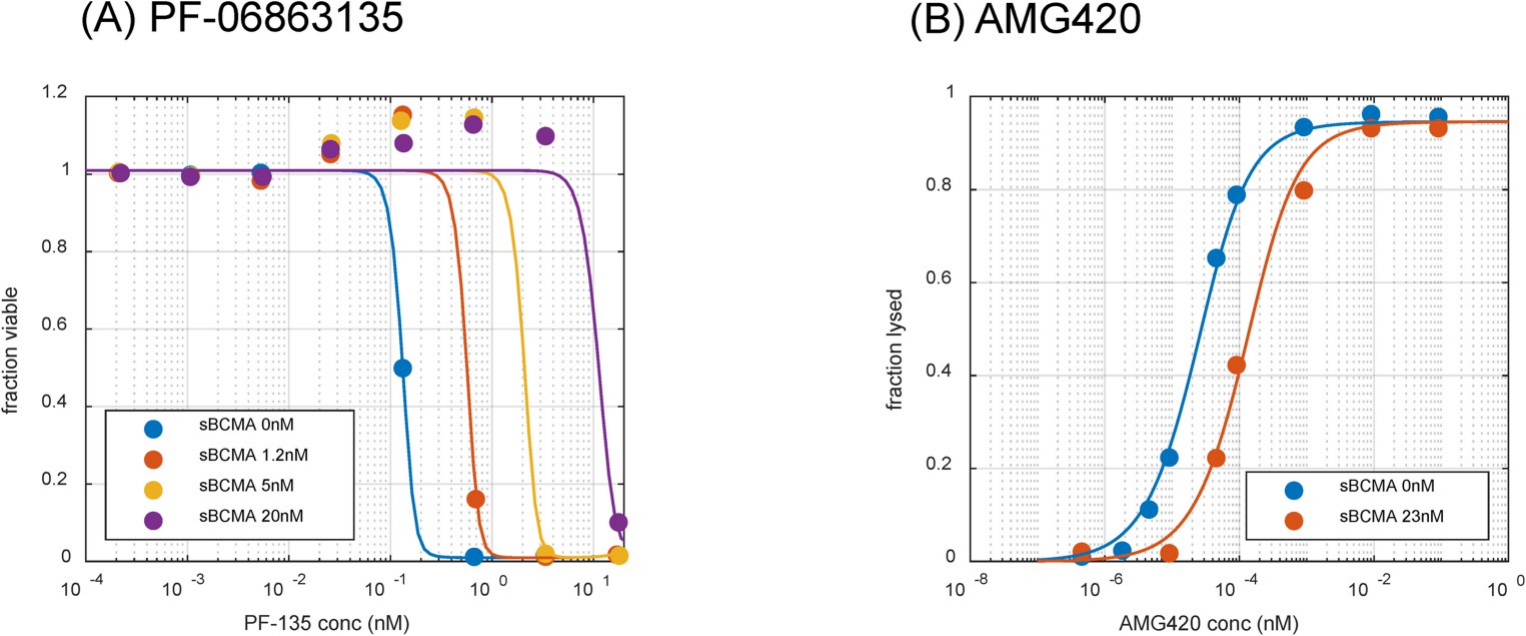
Bispecific TCE binding model-based characterization of in vitro cytotoxicity assay of TCEs. Overlay of experimental observations and model simulation of the relationship between concentrations and cytotoxicity for (A) PF-06863135 (an IgG format, 150 kDa) and (B) AMG420 (a BiTE format, 54 kDa) in the absence or presence of various concentrations of shed BCMAs. In each panel, symbols and lines represent mean observed data and model predictions, respectively. The experimental data were from Panowski et al [29] and Hipp et al [30]. sBCMA: shed BCMA.

A bispecific TCE binding model for immune synapse formation was developed and applied to in vitro cytotoxicity assays of PF-06863135 and AMG420 reported in the literature [29,30]. The detailed descriptions of the mathematical model can be found in the Materials and Methods section as well as in S1 Text. A schematic description of model structure is shown in Fig 1A. The overlays of model-simulated and experimental data of concentration-cytotoxicity relationship after treatment of PF-06863135 and AMG420 in the absence or presence of various concentrations of shed BCMAs are shown in Fig 2A and 2B respectively. The model parameters obtained from the literature or estimated based on observed data are summarized in Table 1. The developed mechanistic bispecific binding model reasonably and quantitatively characterized the concentration-dependent cytotoxicity of tumor cells for both PF-06863135 and AMG420 over a wide range of TCE concentrations while accounting for a concentration-dependent inhibitory effect of shed BCMAs. The binding affinity to shed BCMAs and cytotoxicity-related parameters were estimated using fixed parameters of binding affinities to tumor cell surface BCMAs and CD3s, as well as their expression levels in in vitro systems. It was necessary to estimate baseline shed BCMA expression for the PF-06863135 assay to quantitatively delineate the concentration-dependent inhibitory effect of shed BCMAs, which suggested multiple myeloma cells used in the literature may have produced a small amount of shed BCMAs in the in vitro assay system. This estimation was not performed for AMG420 assay because only one concentration level of shed BCMAs (and absence of shed BCMAs) was tested; therefore, binding affinity to shed BCMAs and its baseline expression cannot be separately estimated. Nevertheless, an attempt was made to estimate the binding affinity to shed BCMAs by AMG420 by assuming the same baseline level of shed BCMAs (0.321 nM) from the PF-06863135 assay, resulting in merely an approximately 10% change from the original parameter estimate, which suggests that a low level of shed BCMAs would not cause substantial change in binding affinity estimation in the AMG420 assay. Taken together, our model analysis indicated that AMG420 had better selectivity to BCMAs over shed BCMAs (0.1 nM to BCMAs and 5.36 nM to shed BCMAs) compared with PF-06863135 (0.04 nM to BCMAs and 0.0319 nM to shed BCMAs). Less significant influence of shed BCMAs was consistently observed in a cytotoxicity assay of AMG420 using NCI-H929 cells as well [30]. While maximum tumor killing rate by immune synapse (kkillmax) was very similar between PF-06863135 and AMG420 (2.38 and 2.93 1/nM/day), the immune synapse numbers per tumor cell at half maximum tumor killing rate (kkill50) differed substantially (12.0 and 0.0229), possibly due to interlaboratory experimental differences. The average number of trimers per tumor cell was calculated for PF-06863135 and AMG420 and plotted against cytotoxicity effects in in vitro assays as shown in Fig A in S1 Text. The degree of cytotoxicity correlated with the average number of trimers per tumor cell regardless of the levels of added shed BCMAs. The average number of trimers per tumor cell corresponding to 50% lysis were approximately 9 and 0.006 for PF-06863135 and AMG420, respectively, which were very close to the kkill50 values estimated from the respective in vitro cytotoxicity assays.

**Table 1:** Summary of parameters for in vitro cytotoxicity assays for PF-06863135 and AMG420.

Parameter	Unit	Description	Value (%RSE)		Source
			PF-06863135	AMG420	
kon	1/nM/day	association rate constant to target, CD3 and shed target	86		[31]
koff	1/day	dissociation rate constant to target, CD3 and shed target	calc from kon and kd		
kd, target	nM	equilibrium dissociation constant to target	0.04	0.1	*PF-06863135 [29]
kd, CD3	nM	equilibrium dissociation constant to CD3	17	20	*AMG420 [32]
kd, shed target	nM	dissociation rate constant to shed target	0.0319 (45.0)	5.36 (14.1)	estimated from in vitro data
CD3 exp	nM	baseline CD3 expression level	0.0415	0.0498	PF-06863135, E:T = 5:1 [29] AMG420, E:T = 6:1 [30]
BCMA exp	nM	baseline BCMA expression level	0.00105	0.00105	
sBCMA exp	nM	baseline shed BCMA expression level before addition	0.321 (56.2)	-	
kg	nM/day	tumor growth rate	0.291 (3.9)		estimated from data in literature [33]
kkillmax	1/nM/day	maximum tumor killing rate by immune synapse	2.38 (80.8)	2.93 (6.7)	
kkill50	nM	immune synapse number per tumor cell at half maximum tumor killing rate	12.0 (33.7)	0.0229 (12.5)	
hill	-	hill coefficient for tumor killing	5.89 (189)	1	
Baseline fraction	-	baseline tumor fraction	1.01 (1.4)	1	
proportional error			0.0732	0	
additive error			0.00593	0.0250	

*source for both kd,target and kd,CD3

### 2.2 Establishment of a molecular size-dependent two-pore biodistribution model for different sizes of TCE proteins in mice

**Fig 3:**
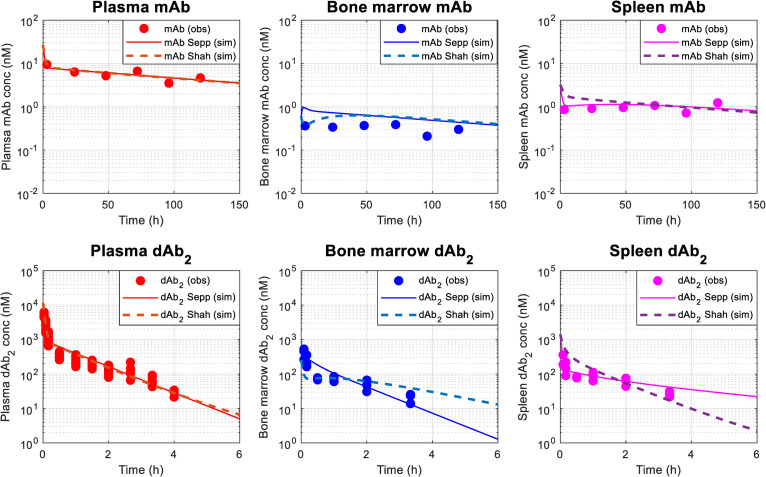
Simplified two-pore theory model-based characterization of biodistribution of different molecular sizes of antibody fragments in mice. Overlay of experimental and model simulation of concentration-time profiles of (A) mAb (150 kDa) and (B) domain antibody (dAb_2_, 25.6 kDa) in plasma, bone marrow, and spleen in mice after a single intravenous administration of mAb at 3.8 μg or dAb_2_ at 10 mg/kg in mice. In each panel, symbols represent experimental data from Shah et al [23] and Sepp et al [25]. Solid and dashed lines represent model predictions under lymphatic flow rates reported by Sepp et al [25] and Shah et al [23], respectively.

The molecular size-dependent physiologically based two-pore theory model was adapted from literature [24–26] and then simplified in the following steps to apply to the TCE model: 1) tissues except for bone marrow, spleen, and lymph node were lumped into one other tissue, 2) endosomal compartments were removed, 3) tissue vascular spaces were lumped into central blood compartment assuming quasi-equilibrium, and 4) biodistribution processes between central blood and tissue interstitial spaces were simplified under quasi-steady state assumption. More detailed descriptions are found in Materials and Methods as well as S1 Text. The schematic model structure of the two-pore theory biodistribution model is depicted in Fig 1B. The schematic model simplification flow of the two-pore theory biodistribution model is depicted in Fig B in S1 Text. The model parameters used and estimated are summarized in Tables A and B in S1 Text. The overlay of experimental and simplified model simulated concentration-time profiles of monoclonal antibody (mAb, 150 kDa) and domain antibody (dAb_2_, 25.6 kDa) in plasma, bone marrow, and spleen in mice are described in Fig 3. The comparison of the original and simplified two-pore theory models are described in Fig C in S1 Text. Without considering endosomal degradation and FcRn-mediated recycling in endosomal compartments, which were incorporated in the original model, the simplified two-pore model reasonably delineated the experimental biodistribution data of very different sizes of proteins in mouse bone marrow and spleen in a manner comparable with the original model. When an impact of tissue lymphatic flow rates was compared using values reported by Sepp et al [25] and Shah et al [23], the former captured experimental biodistribution data better, especially for domain antibody concentrations in bone marrow and spleen, since Shah et al assigned 0.2% of plasma flow rates as lymphatic flow rate, while Sepp et al directly estimated lymphatic flow rates based on tissue distribution of dAb_2_ in mice.

### 2.3 Translation and verification of a molecular size-dependent two-pore theory biodistribution model for TCEs in patients

**Fig 4:**
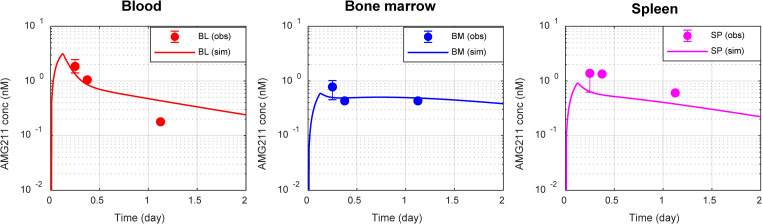
Translated two-pore theory model-based characterization of biodistribution of TCEs in patients. Overlay of experimental positron emission tomography imaging data and model simulation of concentration-time profiles of AMG211 (a carcinoembryonic antigen-targeting TCE, a BiTE format, 54 kDa) in blood, bone marrow, and spleen in patients after an intravenous infusion of [^89^Zr]AMG211 at 37 MBq/200 μg with cold AMG211 at 1800 μg for 3 hours. The translated two-pore theory model was integrated with a T cell extravasation and biodistribution component followed by mechanistic binding of TCEs to T cells. Symbols are observed mean for blood and median for tissues (n = 4). Lines represent model predictions. The experimental data were from Moek et al [28].

The simplified two-pore model was translated from mice to humans by physiologically scaling up plasma and lymphatic flow rates as well as tissue volumes. To characterize bispecific TCE binding dispositions, the model was further extended to incorporate the T cell extravasation model characterized by Khot et al [34]. The T cell extravasation model was employed after simplification in transmigration processes followed by physiological scaling up. The model comparability before and after the simplification of transmigration processes in mice is shown in Fig D in S1 Text. The developed model was verified using clinical PET imaging data of AMG211, a CEA-targeting TCE in a BiTE format (54 kDa), where biodistribution of AMG211 to tissues including bone marrow and spleen was evaluated [28]. The model parameters are summarized in Tables 2 and 3. The overlays of experimental and translated model-simulated concentration-time profiles of AMG211 in blood, bone marrow, and spleen in patients are shown in Fig 4. Having estimated only systemic clearance (CL) of AMG211, the biodistribution of AMG211 into bone marrow and spleen was reasonably characterized, suggesting that the developed physiologically-based two-pore theory biodistribution model can be applied translationally to characterize the biodistribution of different molecular sizes of TCEs followed by bispecific TCE binding in tissues of interest. When compared with a model without T cell biodistribution dynamics, the developed model better predicted the biodistribution of AMG211 into spleen in patients (Fig E in S1 Text).

**Table 2:** Summary of parameters related to bispecific binding, two pore theory and systemic elimination of shed BCMAs, AMG420, AMG211 and PF-06863135 in patients.

Category	Parameter	Unit	Description	Value (%RSE)				Source
				Shed BCMA	AMG420	AMG211	PF-06863135	
Binding-related parameters	target	-	tumor-associated antigen target	-	BCMA	CEA	BCMA	
	kon	1/nM/day	association rate constant	86				[31]
	koff	1/day	dissociation rate constant	calc from kon and kd				
	kd, target	nM	equilibrium dissociation constant	-	0.1	5.5	0.04	*PF-06863135 [29] *AMG211 [28]
	kd, CD3	nM	equilibrium dissociation constant	-	20	310	17	*AMG420 [32]
	kd, shed target	nM	dissociation rate constant	-	5.36	-	0.0319	estimated from in vitro data [29, 30]
Two-pore theory-related parameters	MW	Da	molecular weight	5000	54000		150000	
	a_e_	nm	Stokes-Einstein radius	1.29	3.24		4.81	calc by MW
	σ_vL_	-	vascular reflection coefficient of large pore	0.0157	0.0865		0.18	calc by MW
	σ_vS_	-	vascular reflection coefficient of small pore	0.305	0.902		0.998	calc by MW
	A_L_/A_0L_	-	fractional accessible large pore size	0.83	0.524		0.349	calc by MW
	A_S_/A_0S_	-	fractional accessible small pore size	0.21	0.00267		9.28E-07	calc by MW
	r_L_	nm	large pore size	22.85				[24]
	r_S_	nm	small pore size	4.44				[24]
	α_L_	-	fractional hydraulic conductance of large pore	0.042				[24]
	α_S_	-	fractional hydraulic conductance of small pore	0.958				[24]
	xj	-	coefficient for isogravimetric lymph flow	0.38				[24]
	$\Delta P-\bar{\sigma_{a}}∙\Delta \pi$	mmHg	starling force	1				[24]
Systemic elimination	CL	L/day/kg	plasma clearance	1.55	0.540 (11.5)	0.164 (52.1)	0.00372	PF-06863135 [31], shed BCMA [35] and estimated for others
	Proportional error			-	0.458	0.330	-	

*source for both kd,target and kd,CD3

**Table 3:** Summary of physiological parameters in patients.

Tissue	Total volume (L/kg)	Plasma volume (L/kg)	Interstitial volume (L/kg)	Plasma flow (L/day/kg)	Lymphatic flow (L/day/kg)	Lymphatic reflection coefficient	T/tumor cell transmigration rate (L/day/kg)	BCMA		CD3		T cell degradation rate (1/day)	shed BCMA (nM)
								conc (nM)	expression (#/cell)	conc (nM)	expression (#/cell)		
plasma	0.044	-	-	61.5	-	-	-	4.87E-05	12590	0.393	100000	-	100
bone marrow	0.0423	0.0032	0.0266	0.88	0.0155	0.2	11.4	8.30E-01		3.66		-	calc by model
spleen	0.0031	0.0004	0.00062	2.14	0.00018	0.2	1.88	3.49E-02		98.26		0.03	calc by model
other tissue	0.818	0.021	0.133	58.5	0.240	0.2	89.2	3.82E-04		2.21		0.03	calc by model
lymph node	0.00386	-	-	-	0.256	-	-	1.47E-02		64.5		-	calc by model
Source	[23, 36]	[23]	[23]	[23]	calc by [23, 25]	[23]	[34]	calc by [37–39]	[40]	calc by [38, 41–43]	[11]	[44]	[45]

### 2.4 Application of a translational platform model to BCMA-targeting TCEs in multiple myeloma patients

**Fig 5:**
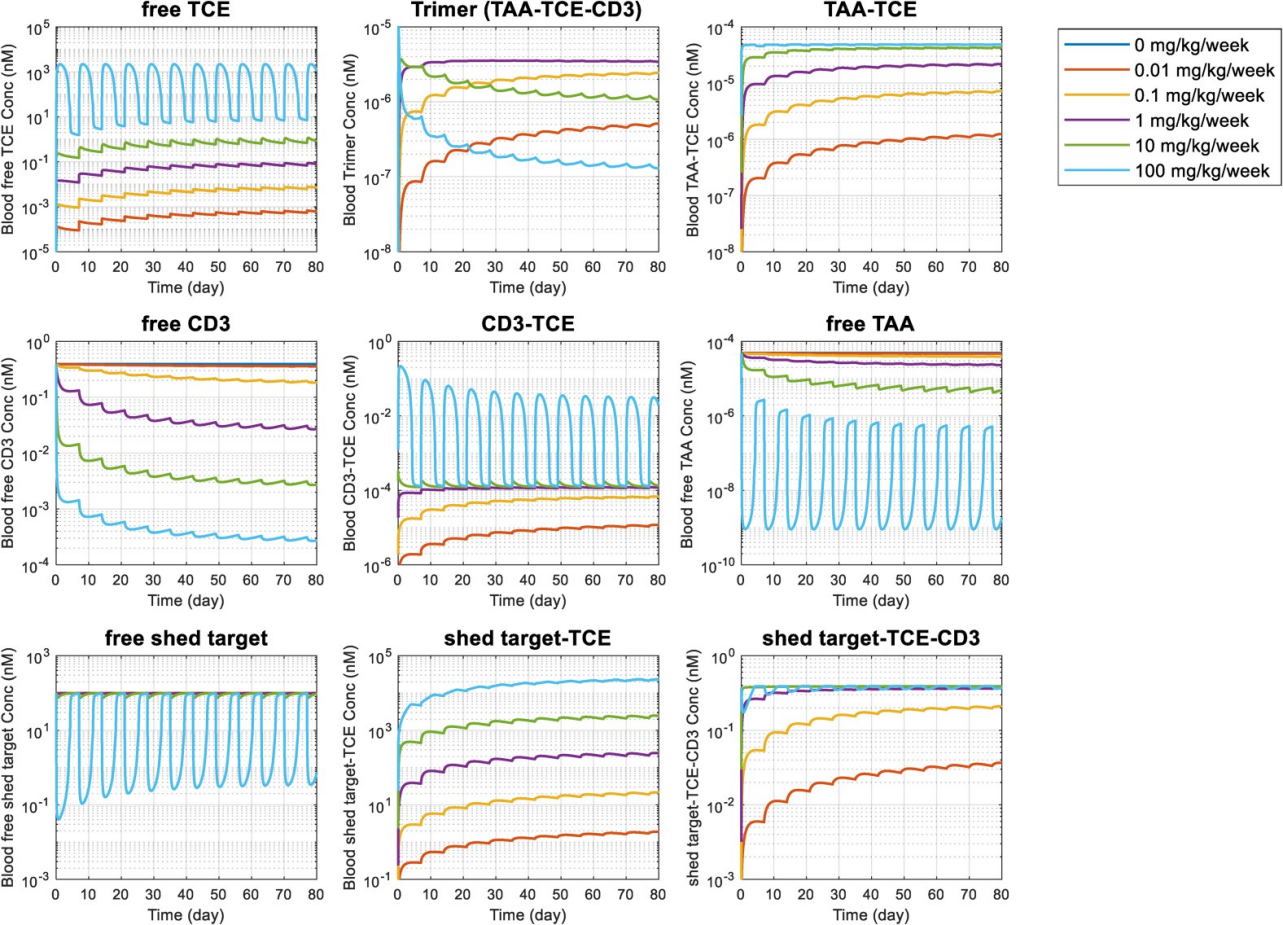
Platform TCE model simulation of concentration-time profiles of variable species relating to bispecific TCE binding in blood after subcutaneous administration of PF-06863135 (an IgG format, 150 kDa) at 0, 0.01, 0.1, 1, 10, and 100 mg/kg with a once weekly dose schedule in multiple myeloma patients. Simulation results were plotted for free TCEs, TAA-TCE-CD3 trimers (immune synapse), TAA-TCE dimers, free CD3s, TCE-CD3 dimers, free TAAs, free shed targets, shed target-TCE dimers, and shed target-TCE-CD3 trimers in blood.

The verified translated model was calibrated for clinical stage BCMA-targeting TCEs, PF-06863135 in an IgG format at molecular weight of 150 kDa and AMG211 in a BiTE format at molecular weight of 54 kDa. A schematic model structure of a physiologically-based platform model for TCEs is shown in Fig 1C. The model parameters used and estimated are summarized in Tables 2 and 3. Biodistribution of tumor cells (multiple myeloma cells) was modeled in a similar way to that of T cells. Concentration of BCMAs, which is expressed on multiple myeloma cells in blood and different tissues, is calibrated based on literature information [37–40]. The platform TCE model simulation of baseline concentration-time profiles of free BCMAs, CD3s, and shed BCMAs in blood, bone marrow, spleen, and other tissue compartment without a TCE treatment are depicted in Fig F in S1 Text. It was confirmed that the E:T ratio in bone marrow calculated by the present model (0.555, Table C in S1 Text) was well within the range of reported E:T ratios (geomean: 1.81, geometric SD: 3.97, n = 43) [46]. Systemic clearance (CL) of AMG420 was estimated against published clinical PK data [47], while general antibody CL was employed for PF-06863135 [31]. The overlay of observed and model simulated plasma concentration time profiles of AMG420 is shown in Fig G in S1 Text. The model-predicted time profiles of each species (free TCEs, free TAAs, free CD3s, free shed targets, dimers, and trimers) after treatment with PF-06863135 and AMG420 are shown in Fig 5 and Fig H in S1 Text, respectively. The simulation results indicated a dose-dependent monotonic increase of free TCEs and dimer species (TAA-TCE, CD3-TCE, shed target-TCE). On the other hand, a nonmonotonic dose response was predicted for immune synapse formation, where a dose-dependent increase was observed up to approximately 1 mg/kg/week of PF-06863135, while immune synapse levels at 10 and 100 mg/kg/week were lower than at 1 mg/kg/week at the steady state. A similar nonmonotonic relationship of dose to immune synapse formation was predicted for AMG420 as well (Fig H in S1 Text). These predicted nonmonotonic dose responses in immune synapse formation were consistent with two previous publications on bispecific TCE modeling [14,18].

**Fig 6:**
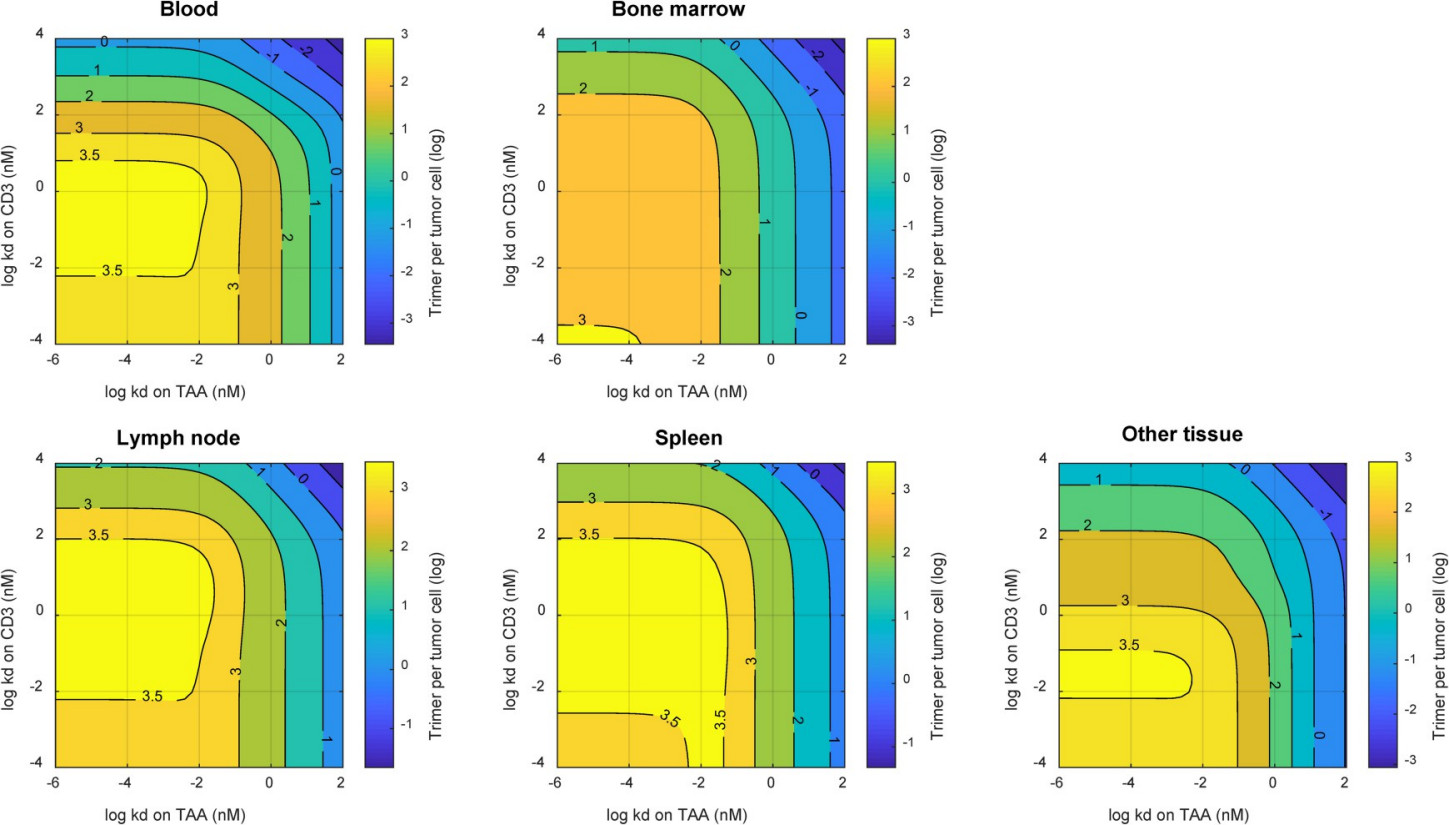
Platform TCE model simulation of two-dimensional contour plots for the effects of TCEs’ equilibrium dissociation constants (kds) on TAAs and CD3s on immune synapse (TAA-TCE-CD3 trimer) formation in blood, bone marrow, lymph node, spleen, and other tissue compartment after subcutaneous TCE (an IgG format, 150 kDa) administration once weekly in patients. Other parameters were the same as those for PF-06863135.

The developed model of TCEs was further explored using sensitivity analysis to explore its potential to aid in the protein design of TCEs. The results of local sensitivity analyses are shown in Figs I and J in S1 Text. Immune synapse formation in bone marrow was sensitive with a positive correlation for both PF-06863135 and AMG420 to the following parameters: baseline expression of TAAs and CD3s in bone marrow and an equilibrium dissociation rate coefficient (kd) to shed targets. Immune synapse formation in bone marrow was sensitive with a negative correlation to the following parameters: baseline expression of shed targets, kd to TAAs and CD3s, and an elimination rate of shed targets. Systemic clearance was found to be a negatively sensitive parameter only to AMG420, representing faster elimination of AMG420 due to a lack of an FcRn binding domain. Further sensitivity analysis was performed with a wider range of parameter space for key parameters identified from the local sensitivity analysis: expression levels of and kds to TAAs, CD3s, and shed targets. The results are shown in Fig 6 as well as Figs K and L in S1 Text. Monotonic relationships were confirmed for expression levels of and kds to TAAs, CD3s, and shed targets, except for a kd to CD3s. These results highlight the importance of the inhibitory relationship between TAAs and shed targets, suggesting the selectivity between TAAs and shed targets is one of key factors to maximize immune synapse formation, which enhances anti-tumor effects. Furthermore, an intriguing theoretical “sweet spot” was implied for binding affinity to CD3s (Fig 6). In blood, for example, the predicted immune synapse level was monotonically increased when the kd to CD3s was decreased down to around 1–10 nM, while the immune synapse level started to decrease when the kd was further lowered below 0.01 nM. On the other hand, a one-compartmental bispecific binding model that consists of only one compartment did not imply the nonmonotonic effect of CD3 binding affinity on immune synapse formation (Fig M in S1 Text).

**Fig 7:**
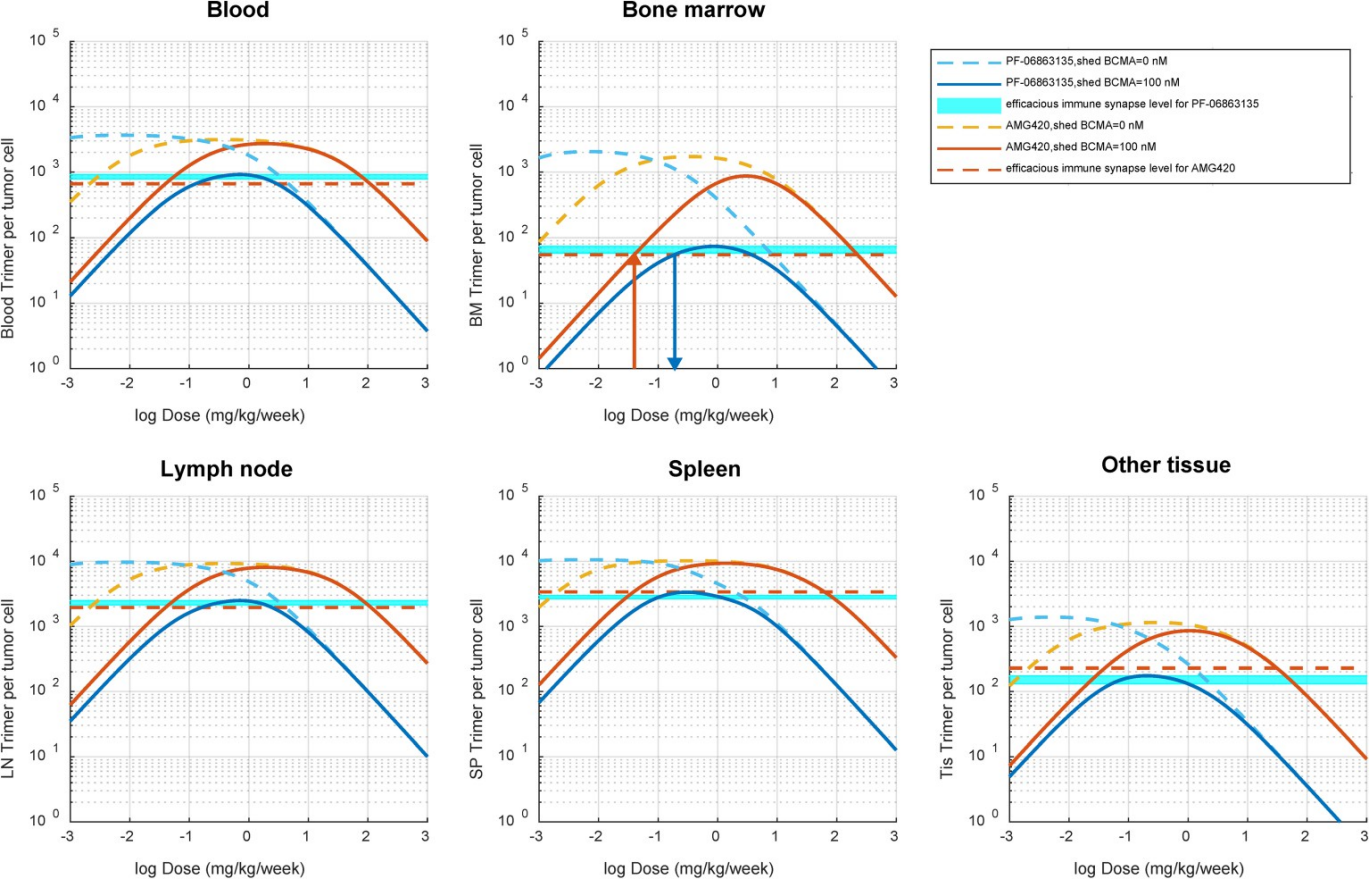
Platform TCE model-based simulation of dose-to-immune-synapse-level relationship for PF-06863135 (an IgG format, 150 kDa) and AMG420 (a BiTE format, 54 kDa) in multiple myeloma patients. The simulation was performed for immune synapse levels in blood, bone marrow, lymph node, spleen, and other tissue compartment at a wide range of TCE dose levels (0.001 to 1000 mg/kg/week) in the absence or presence (100 nM) of shed BCMAs in multiple myeloma patients. PF-06863135 was administered once weekly in subcutaneous injections and AMG420 was administered under a continuous intravenous infusion for 28 days. The horizontal light blue shade and red dashed line represent simulated immune synapse levels at the reported efficacious dose ranges of PF-06863135 (215 to 1000 mg/kg/week) and AMG420 (0.04 mg/kg/week), respectively. The red vertical arrow represents the predicted immune synapse level in bone marrow at the reported efficacious dose level of AMG420 (0.04 mg/kg/week), and the blue vertical arrow represents the projected dose level of PF-0686135 (0.191 mg/kg/week) at the corresponding immune synapse level projected from the efficacious dose of AMG420 in patients.

The platform model-simulated relationship of the dose of different molecular sizes of TCEs (PF-06863135 and AMG420) to immune synapse formation, in the absence or presence of shed BCMAs, is depicted in Fig 7. In absence of shed BCMAs, both PF-06863135 and AMG420 reached a similar level of maximum immune synapse formation in all of the tissues predicted, while PF-06863135 achieved it at lower weekly doses, which reflects its longer systemic half-life as a full IgG format. On the other hand, in the presence of shed BCMAs (100 nM) at an average concentration observed in multiple myeloma patients [45], the maximum level of immune synapse formation was greatly attenuated for PF-06863135, which reflects a narrower selectivity window between tumor cell surface BCMAs and shed BCMAs estimated from the in vitro cytotoxicity assay. This prediction emphasizes the importance of selectivity of TCEs to TAAs over shed targets, which is consistent with the finding from the sensitivity analysis. It was noted that the predicted number of immune synapses per tumor cell was lower in bone marrow compared with other compartments. This is due to the combination of two factors: 1) higher shed BCMAs, and 2) the lower E:T ratio in bone marrow as shown in Table C in S1 Text. At the reported clinically efficacious dose of AMG420 (400 μg/day or 0.04 mg/kg/week for 28 days continuous infusion) (36), the immune synapse level was predicted to be 55.0/tumor cell in bone marrow. Without any further calibration, the corresponding dose level of PF-06863135, where the immune synapse level was at 55.0/tumor cell in bone marrow, was estimated to be 0.191 mg/kg/week for a once weekly subcutaneous injection. This projection was in extraordinary agreement with the reported clinically efficacious dose of PF-06863135 (0.215 to 1 mg/kg, once weekly subcutaneous injection) [48]. The projected immune synapse level in bone marrow ranged 58.3 to 73.9/tumor cell at the reported efficacious dose of PF-06863135, indicating that the projected immune synapse levels in bone marrow were similar between PF-06863135 and AMG420 at their reported efficacious dosing regimens in multiple myeloma patients.

## 3 Discussion

A quantitative computational modeling framework was developed for TCEs integrating molecular size-dependent two-pore biodistribution theory, extravasation of T cells and tumor cells, as well as mechanistic bispecific binding of TCEs to bridge tumor cells and T cells in the presence of shed targets. Although the current model consists of blood, bone marrow, lymph node, and spleen, reflecting the major tissues of interest for TCE treatments in multiple myeloma patients, the framework can be easily extended to a whole body scale in a full PBPK model. It is also possible to introduce a solid tumor compartment by adapting a molecular size-dependent tumor disposition model [20]. Since there are considerable challenges in applying TCEs to solid tumor indications, the quantitative modeling framework could potentially provide useful insights for molecular design of TCEs considering unique physio-pathology associated with solid tumor micro-environments as an extension of the present study.

As demonstrated by the modeling results, this modeling framework can be translationally scaled from mice to humans to describe biodistribution of protein therapeutics while considering species-specific physiological parameters (e.g., plasma and lymphatic flows, plasma and tissue volumes) as well as abundance of targets, T cells, and shed targets. To the best knowledge of the authors, this is the first study to demonstrate that tissue distribution of TCEs in patients could be translationally predicted by a two-pore theory model combined with T cell abundance in tissues. The incorporation of a T cell transmigration/biodistribution mechanism improved the characterization of TCE distribution into spleen (Fig E in S1 Text). This mechanism may also be beneficial in possibly extending this model to incorporate CAR-T cell therapies. In light of the translational applicability of the two-pore theory, the model was further extended for biodistribution of shed targets and their subsequent competitive interactions with TCEs. Betts and colleagues developed a TCE model with shed target interactions in the central blood compartment, but the model did not account for those interactions in the tumor [14]. Our framework is valuable in predicting shed target concentrations at a site of action only from the circulating level of shed targets and their molecular weight. The developed framework indicated that competitive interactions of TCEs between membrane surface targets and their shed targets at a site of action is critical to determine the extent of immune synapse formation in the tumor as shown in Fig L in S1 Text. While there are uncertainties in predicting biodistribution of shed targets, it is important to note that the two-pore theory biodistribution model has encouragingly broad applicability not only to albumin and antibody fragments but also to cytokines (such as IL-2, IL-10, IL-11, etc) and other therapeutic proteins (such as IGF-1, hGH, EPO, etc) [26]. Thus, an important future research direction could be characterizing the diffusion of proteins in tissue interstitial spaces with respect to its charge, isoelectric point, and glycosylation status [24,26].

The developed modeling framework highlighted that the key feature of TCE therapeutics in immune synapse formation is a function of binding affinities as well as expression levels of TAAs, CD3s, and shed targets (Fig 6 as well as Figs K and L in S1 Text). Especially the selectivity to tumor cell surface targets over shed targets is one of the most important molecular design parameters when the therapeutic target exhibits considerable shedding. As discussed above, the developed platform modeling framework is useful to project competitive interactions of TCEs to targets and shed targets at a site of action by employing the experimental assay results for binding selectivity estimation.

The developed model implied the existence of a “sweet spot” for binding affinity to CD3s, suggesting that too-tight CD3 binding may not necessarily provide additional benefit on immune synapse formation (Fig 6). A corresponding experimental observation has been reported in which higher binding affinity to CD3s negatively affected the systemic exposure of TCEs as it shifted distribution of TCEs away from the tumor tissue to T-cell-rich tissues [49]. This observation was supported by an additional modeling exercise in which the implied “sweet spot” of CD3 binding affinity disappeared when the T cell expression level was set uniform across all the tissues and blood, suggesting that tissue-dependent CD3 expression gradients affected the biodistribution and retention of TCEs and immune synapse formation. More importantly, a one-compartmental bispecific binding model could not reproduce the nonmonotonic effect of CD3 binding affinity on immune synapse formation (Fig M in S1 Text), suggesting the need of a relevant in vivo system or multicompartment mechanistic computational model rather than a simple in vitro cytotoxicity assay to aid the optimization of CD3 binding affinity during a lead optimization stage of drug development.

The developed model was further utilized to gain a better understanding of the amount of immune synapse required for distinct molecular sizes and formats of TCEs, namely PF-06863135 in an IgG format and AMG420 in a BiTE format. The difference in molecular sizes and protein formats seemingly causes a substantial difference in PK and tissue distribution disposition. Nevertheless, the final platform model predicted a considerable agreement in the immune synapse level in bone marrow (~55/tumor cell) at the reported respective efficacious dose levels of PF-06863135 (0.215 to 1 mg/kg under once weekly subcutaneous injections [48]) and AMG420 (400 μg/day or 0.04 mg/kg/week under a continuous intravenous infusion for 28 days [47]) in multiple myeloma patients (Fig 7). The predicted number of immune synapses was low (~55/tumor cell) compared with average BCMA expression on multiple myeloma cells (~12590/tumor cell) [40]. This result suggests that model-based simulation of immune synapse levels at a site of action can be a good strategy to translationally predict the efficacious dose of TCEs in patients. The confidence in the model predictions can be strengthened in case clinical benchmarking insight is available from precedented molecules regardless of molecular sizes or formats since the required information is limited to the molecular weight of TCEs, binding affinity to targets, CD3s, and shed targets, as well as target and shed target expression levels. On the other hand, it should be noted that the model developed in the present study has two limitations: 1) TCE format-dependent binding avidity in crosslinking: Although the available data are partial, archetype antibody formats (i.e., IgG-like, diabodies, BiTEs) likely have different intrinsic potencies [50]. As our model does not currently account for this effect, this is certainly the area of further model improvement; and 2) Dynamics of T cells, tumor cells, and shed targets: It has been reported that activated T cells were migrated from blood and proliferated in tissues after treatments of blinatumomab in acute lymphoblastic leukemia (ALL) patients [17]. In addition, the depletion of multiple myeloma cells is correspondent with decrease of shed BCMAs in multiple myeloma patients [47]. These dynamic changes in systems will impact the immune synapse formation and should be carefully incorporated into the modeling framework in the future, which will better address possible pathophysiological and pharmacodynamic alterations induced by TCE treatments in patients.

Another important observation from the current model is notably different potencies between PF-06863135 and AMG420 observed in in vitro cytotoxicity assays despite the good agreement in projected immune synapse formation in bone marrow using reported clinically efficacious doses in patients. The in vitro cytotoxicity-related parameters of immune synapses per tumor cell (kkill50) varied greatly (12.0 for PF-06863135 and 0.0229 for AMG420) even when using the same tumor cells, namely L-363 myeloma cells. Notably, the estimated kkill50 value of PF-06863135 from the in vitro cytotoxicity assay was close to the immune synapse number per tumor cell at the efficacious dose in patients (~55), while that of AMG420 in vitro was far off. The possible reason behind this mismatch might be the experimental conditions and sensitivity difference between experiments since these in vitro assay results were collated from different publications describing experiments likely performed in different laboratories. It has been hypothesized that the source of T cells can generate variability in the results of an in vitro cytotoxicity assay of TCEs under co-incubation of tumor cells and effector cells [51]. A large sensitivity difference also has been observed between NCI-H929 and L-363 cells when treated with AMG420 in vitro [30]. Ideally, in vitro assays performed in a controlled setting may provide more quantitative insights. Nevertheless, in vitro assays in the presence of shed targets provided useful information to estimate the binding affinity/selectivity to shed targets. In addition, Jiang and colleagues reported that in vitro cytotoxicity assays were found to predict clinical efficacy of blinatumomab (an anti-CD19-targeting TCE in a BiTE format) in ALL patients [12] by introducing attenuated T cell cytotoxicity (one-third of in vitro value) while taking into account immunosuppressive environment in bone marrow in ALL patients compared with in vitro setting. In vitro to in vivo extrapolation of TCE efficacies is obviously an area that needs further investigation.

In conclusion, a quantitative computational framework for TCE-mediated immune synapse formation in patients was developed by integrating the two-pore theory based biodistribution model of different molecular sizes of proteins and T cell extravasation model followed by mechanistic bispecific binding in the presence of shed targets. The developed framework demonstrated the translational predictability of biodistribution of a TCE into bone marrow and spleen in patients, which was supported by PET imaging in the clinic. The model framework was particularly useful to explore optimal molecular design parameters of TCEs across a variety of molecular sizes and formats while considering tissue distribution as well as mechanistic bispecific binding of TCEs to tumor cell surface TAAs and T cells under competitive inhibitory interactions against shed targets. The model highlighted the importance of selectivity to targets on tumor cells over shed targets. The developed modeling framework can quantitatively guide how much selectivity is required in binding affinities in context of expression levels of TAAs and shed targets at a site of action. Moreover, the model implied the existence of a “sweet spot” of CD3 binding affinity, which was not able to be predicted by a one-compartment model, emphasizing the usefulness of multi-tissue and multi-scale platform models. The model-simulated immune synapse levels in bone marrow were found comparable between clinical stage BCMA-targeting TCEs, namely PF-06863135 and AMG420, at the reported efficacious dose and dose schedule, suggesting the developed model framework is powerful at leveraging clinical benchmarking insights regardless of the difference in molecular sizes and formats as well as their associated distinct dispositions in pharmacokinetics and tissue distribution. This framework can be applied to other targets beyond multiple myeloma to provide a quantitative means of molecular design and clinical benchmarking in order to facilitate the model-informed best-in-class TCE discovery and development.

## 4 Materials and methods

### 4.1 A bispecific TCE binding model for in vitro cytotoxicity assay in the presence of shed targets

Bispecific TCE binding was characterized by a sequential mechanistic binding kinetics model. A schematic description of the model structure is shown in Fig 1A. Differential equations for bispecific binding reactions are shown in S1 Text. The mechanistic binding model was applied to in vitro cytotoxicity assays of PF-06863135 and AMG420 reported in the literature [29,30]. A series of concentrations of PF-06863135 were co-incubated with luciferase-labelled L-363 myeloma cells and human CD3+ T cells at an E:T ratio of 5:1 (25000 T cells and 5000 myeloma cells) in the absence or presence of shed BCMAs at 1.2, 5, and 20 nM. After 2 days of incubation, viability of myeloma cells was assessed by luminescence measurement [29]. Likewise, a series of concentrations of AMG420 were co-incubated with L-363 cells and T cells at an E:T ratio of 6:1 in the absence or presence of shed BCMAs at 115 ng/mL (23 nM). The depletion of target cells was measured by flow cytometry analysis after 24 h of incubation [30]. Temporal dynamics of TAA concentrations were accounted for in context of tumor growth and immune synapse-mediated tumor killing. Parameters used and estimated for in vitro cytotoxicity results are provided in Table 1. Target and CD3 expression levels were calculated from assay conditions in the literature [29,30].

### 4.2 Simplification of a molecular size-dependent two-pore theory biodistribution model for different sizes of proteins in mice

The biodistribution of different molecular sizes of TCEs and shed targets was delineated using the two-pore theory biodistribution model adapted from the literature [24]. A schematic description of two-pore theory biodistribution model structure is shown in Fig 1B. According to the two-pore theory, proteins are transported from vascular spaces to interstitial spaces via two groups of pores (small pores: ~4.44 nm, large pores: ~22.9 nm, with fractional hydraulic conductance at 0.958 and 0.042, respectively) by diffusion and convection. Equations to determine molecular size-dependent transport processes under the two-pore theory can be found in S1 Text. The original full-tissue two-pore model was simplified. Detailed model simplification steps are described in S1 Text. A schematic model simplification flow is depicted in Fig B in S1 Text. The simplified distribution rate constants in-between central blood and interstitial spaces (Q’ and (Q-L)’) were derived by integrating perfusion as well as diffusion and convectional rate constants through small and large pores in each compartment as follows;

$${Q^{\prime }}_{j}=\frac{Q_{j}∙(\sum_{i=L,S}{PS}_{i}+\sum_{i=L,S}J_{i,j})}{{(Q}_{j}-L_{j})+(\sum_{i=L,S}{PS}_{i}+\sum_{i=L,S}J_{i,j})}$$ Eq 1

$${(Q-L)\prime }_{j}=\frac{(Q_{j}-L_{j})∙\sum_{i=L,S}{PS}_{i}}{{(Q}_{j}-L_{j})+(\sum_{i=L,S}{PS}_{i}+\sum_{i=L,S}J_{i,j})}$$ Eq 2

Where Q, (Q-L), PS, and J represent arterial plasma flows, venous plasma flows, permeability surface areas, and convectional flows, respectively. Subscript L, S, and j represent large pores, small pores, and tissue, respectively.

The simplified two-pore biodistribution model was verified using literature information on tissue biodistribution of nonspecific proteins, dAb_2_ (25.6 kDa) and mAb (150 kDa), in mice [23,25]. Parameters used and estimated for in vivo tissue distribution in mice are provided in Tables A and B in S1 Text. Plasma and tissue concentrations of nonspecific mAb and dAb_2_ were evaluated after a single intravenous administration at 3.8 μg and 10 mg/kg, respectively, in mice. Only systemic clearance (CL) was estimated using experimental plasma concentration-time profile data and then tissue concentration profiles were simulated using a set of lymphatic flow rate parameters reported by either Sepp et al [25] or Shah et al [23]. Shah and colleagues assigned 0.2% of plasma flow rates as lymphatic flow rates, while Sepp and colleagues directly estimated lymphatic flow rates based on tissue distribution of dAb_2_ in mice. The model-simulated tissue concentration-time profiles were compared with the experimental results from the two publications.

### 4.3 Translation and verification of a two-pore biodistribution model for TCEs in patients

The simplified and verified two-pore biodistribution model in mice was translationally scaled to humans by replacing the physiological parameters including blood and lymphatic flow rates as well as blood and tissue volumes. The model was further integrated with the T cell transmigration/biodistribution model from literature [34] by employing the similar model simplification for transmigration of T cells from central blood to tissue interstitial spaces via tissue vascular spaces. More details on model translation and T cell transmigration integration processes can be found in S1 Text. Parameters related to bispecific binding, the two-pore theory, and systemic elimination in patients are provided in Table 2. A summary of physiological parameters in patients is provided in Table 3. The translated computational model for bispecific TCEs was verified using literature information on biodistribution of AMG211, a CEA-targeting TCE, in patients [28]. [^89^Zr]AMG211 at 37 MBq/200 μg with cold AMG211 at 1800 μg was intravenously infused to patients for 3 hours. Whole body PET scans were performed at 3, 6, and 24 hours after completion of the infusion. Assuming limited CEA expression in blood, bone marrow, spleen, and lymph node, kon,target was set to 0 and only CL of AMG211 was estimated. Since free Zr accumulates in the non-soft tissue (i.e. mineralized constituents of the bone), but does not specifically accumulate in the marrow compartment [52], contribution of free ^89^Zr to the PET imaging in bone marrow was deemed marginal. Without further parameter optimization, the biodistribution of AMG211 into bone marrow and spleen was simulated by the human model and compared with PET imaging data of AMG211 collected from patients.

### 4.4 Application of a translational platform model to BCMA-targeting TCEs in multiple myeloma patients

The translated and verified computational model was applied to BCMA-targeting bispecific TCEs in multiple myeloma patients. Biodistribution of tumor cells (multiple myeloma cells) was modeled in a similar manner to that of T cells, and more information can be found in S1 Text. The synthesis, elimination, and biodistribution of shed targets (i.e., shed BCMAs) were also incorporated to explicitly account for the competitive interactions against shed BCMAs at sites of action. Shed BCMAs were assumed to be generated in bone marrow, eliminated from blood circulation mainly through renal excretion, and biodistributed to tissues according to the two-pore theory. A schematic description of the final computational platform model structure is shown in Fig 1C. The final model consists of 45 ordinal differential equations, 197 reactions, and 85 parameters. The model parameters used and estimated are outlined in Tables 2 and 3. Binding affinities of PF-06863135 and AMG420 to shed BCMAs were estimated from in vitro cytotoxicity assays and directly incorporated into the model. Clinical pharmacokinetics of AMG420 were calibrated using literature information on concentration-time profiles of AMG420 with a 28-day continuous infusion in multiple myeloma patients. Clearance of PF-06863135 was assumed to be similar to that of a typical antibody [47]. A subcutaneous dosing of PF-06863135 was described by first-order absorption from a dosing compartment to a blood compartment. The absorption rate constant was set to 0.7 day^-1^ to match the reported Tmax range of PF-06863135 (3–7 days) [53]. The bioavailability was set to 1 considering 1) reported subcutaneous bioavailability of antibodies are relatively high (0.5 to 0.7) [54], and 2) there is uncertainty in other PK parameters taken from the literature [31].

The final platform model was used to simulate concentration-time profiles of variable species relating to bispecific TCE binding in blood, bone marrow, spleen, lymph node, and other tissue compartment after administration of PF-06863135 as a once weekly subcutaneous injection and AMG420 with a continuous intravenous infusion for 28 days in multiple myeloma patients. Local parameter sensitivity analyses were performed around the final sets of model parameters for PF-06863135 and AMG420 to identify parameters to which immune synapse formation is sensitive in blood, bone marrow, and lymph node. Additionally, two-dimensional sensitivity analyses were performed to cover wider ranges of key parameters identified from the local sensitivity analysis: binding affinities to and expression profiles of TAAs, CD3s, and shed targets. The relationships between dose of TCEs (0.001 to 1000 mg/kg/week) and immune synapse formation (average from day 56 to 70 for PF-06863135 and average from day 0 to 28 for AMG420) in blood, bone marrow, lymph node, spleen, and other tissue compartment were simulated and compared after treatments of PF-06863135 and AMG420 in the absence or presence of shed BCMAs (100 nM) in multiple myeloma patients. Trimers per tumor cell was selected as a key readout as this readout is less influenced by different levels of tumor cells (i.e., tumor burden).

### 4.5 Model software

The computational model was developed using the Simbiology toolbox of MATLAB R2019a (Mathworks, Natick, MA). The optimization toolbox with fminsearch was used with ode15s for parameter estimations. The local parameter sensitivity analyses were performed by calculating time-dependent sensitivity indices on immune synapse formation in blood, bone marrow, and lymph node with the full dedimensionalization option, and then the indices were integrated throughout the time course.

## Supporting information

S1 Text Supporting methods. **Table A. Summary of parameters related to the two-pore theory and systemic elimination of dAb2 and mAb in mice. Table B. Summary of physiological parameters in mice. Table C. Summary of baseline concentrations of tumor cells, T cells, and shed BCMAs in multiple myeloma patients. Fig A. In vitro TCE cytotoxicity model-simulated relationship between the average number of trimers per tumor cell and fractions viable or lysed of tumor cells of (A) PF-06863135 and (B) AMG420 in the absence or presence of shed BCMAs in in vitro cytotoxicity assays. Fig B. Schematic model simplification flow of the two-pore theory biodistribution model.** (A) The original two-pore theory model explicitly accounted for a detailed tissue distribution process through perfusion, diffusion, and convection through two groups of pores (small and large pores) in a molecular size-dependent manner as well as uptake by vascular endothelial cells followed by endosomal degradation or an FcRn-mediated recycling mechanism in each tissue. (B) Model simplification process in which endosomal degradation and FcRn-mediated recycling mechanisms were removed and biodistribution rate constants in and out of tissue interstitial space (Q’ and (Q-L)’) were derived by integrating plasma flows as well as diffusion and convection rate constants via small and large pores under a quasi-steady state assumption. Tissue vascular spaces were lumped into central blood under a quasi-equilibrium assumption. (C) Simplified two-pore theory biodistribution model. The final model accounts for molecular size-dependent biodistribution between the central blood compartment and tissue interstitial spaces as well as lymphatic recirculation. Q, Q-L, L, PS, J, and Jiso represent arterial plasma flows, venous plasma flows, lymphatic flows, permeability surface areas, convectional flows, and isogravimetric lymph flows, respectively. Subscript BM, SP, LN, Tis, L, and S represent bone marrow, spleen, lymph node, other tissue, large pore, and small pore, respectively. CLup, konFc, koffFc, kdeg, and FR represent a cellular uptake, association and dissociation rate constants to and from FcRn, an endosomal degradation rate constant, and a fraction recycle to vascular spaces, respectively. Q’ and (Q-L)’ represent biodistribution rate constants in and out of tissue interstitial spaces derived under a quasi-equilibrium assumption. **Fig C. Comparison between the original and simplified two-pore theory model-based characterization of biodistribution of different molecular sizes of antibody fragments in mice.** Overlay of experimental and model simulations of concentration-time profiles of (A) mAb (150 kDa) and (B) domain antibody (dAb2, 25.6 kDa) in plasma, bone marrow, and spleen in mice after a single intravenous administration of mAb at 3.8 μg or dAb2 at 10 mg/kg in mice. In each panel, symbols represent experimental data. Solid and dashed lines represent model predictions by the simplified and original model, respectively, under the lymphatic flow rate reported by Sepp et al. The experimental data were from Shah et al and Sepp et al. **Fig D. Comparison between the original and simplified T cell PBPK model-based characterization of biodistribution of T cells in mice.** Overlay of experimental and model simulations of concentration-time profiles of T cells in blood, bone marrow, and spleen in mice after a single intravenous administration of [^51^Cr]-labelled T cells at 10 μCi per animal. In each panel, symbols represent experimental data. Solid and dashed lines represent model predictions by the simplified and original model, respectively. The experimental data were from Khot et al. **Fig E. Comparison between the translated platform model and a model without T cell dynamics on model-simulated biodistribution of AMG211 in patients.** Overlay of experimental positron emission tomography imaging data and model simulation of concentration-time profiles of AMG211 (a carcinoembryonic antigen-targeting TCE, BiTE format, 54 kDa) in blood, bone marrow, and spleen in patients after intravenous infusion of [^89^Zr]AMG211 at 37 MBq/200 μg with cold AMG211 at 1800 μg for 3 hours. Symbols are observed mean for blood and median for tissues (n = 4). Solid and dashed lines represent model predictions by the platform model and a mode without T cell dynamics, respectively. The experimental data were from Moek et al. **Fig F. Platform TCE model simulation of concentration-time profiles of free BCMAs, CD3s, and shed BCMAs in blood, bone marrow, lymph node, spleen, and other tissue compartment without a TCE treatment in multiple myeloma patients. Fig G. Platform TCE model characterization of plasma pharmacokinetics of AMG420 in multiple myeloma patients.** Overlay of experimental observations and model simulation of plasma concentration-time profile of AMG420 under continuous intravenous infusion of AMG420 (BiTE format, 54 kDa) at 400 μg/day for 28 days in patients. Symbols and a line represent individual observed data and model prediction, respectively. The experimental data were from Topp et al. **Fig H. Platform TCE model simulation of concentration-time profiles of various species in blood after continuous intravenous administration of AMG420 (a BiTE format, 54 kDa) at 0, 0.01, 0.1, 1, 10, 100, and 1000 mg/kg/week for 4 weeks in patients.** Simulation results were plotted for free TCEs, TAA-TCE-CD3 trimers (immune synapses), TAA-TCE dimers, free CD3s, TCE-CD3 dimers, free TAAs, free shed targets, shed target-TCE dimers, and shed target-TCE-CD3 trimers in blood. **Fig I. Local parameter sensitivity analysis of the platform model for PF-06863135 (an IgG format, 150 kDa) in patients.** The integrated sensitivity indices of each parameter on immune synapse levels in blood, bone marrow, and lymph node were calculated after subcutaneous injection of PF-06863135 at a 0.5 mg/kg once weekly dose schedule in multiple myeloma patients. The positive sensitivity indices indicate that increase in a parameter value leads to an increase in immune synapse levels. **Fig J. Local parameter sensitivity analysis of the platform model for AMG420 (a BiTE format, 54 kDa) in patients.** The integrated sensitivity indices of each parameter on immune synapse levels in blood, bone marrow, and lymph node were calculated after intravenous administration of AMG420 at 0.4 mg/day as a continuous intravenous infusion for 28 days in multiple myeloma patients. The positive sensitivity indices indicate that increase in a parameter value leads to an increase in immune synapse levels. **Fig K. Platform TCE model simulation of two-dimensional contour plots for the effects on immune synapse (TAA-TCE-CD3 trimer) formation in blood, bone marrow, lymph node, spleen, and other tissue compartments by changing expression level of TAAs on tumor cells and CD3s on T cells after subcutaneous TCE (an IgG format, 150 kDa) administration once weekly at 1 mg/kg in patients.** Other parameters were the same as those for PF-06863135. **Fig L. Platform TCE model simulation of two-dimensional contour plots for the effects on immune synapse (TAA-TCE-CD3 trimer) formation in blood, bone marrow, lymph node, spleen, and other tissue compartments by changing TCE’s equilibrium dissociation constants (kd) for shed targets and expression level of shed targets after subcutaneous TCE (IgG format, 150 kDa) administration once weekly at 1 mg/kg in patients.** Other parameters were the same as those for PF-06863135. **Fig M. One-compartment TCE model simulation of two-dimensional contour plots for the effects on immune synapse (TAA-TCE-CD3 trimer) formation in blood by changing TCEs’ equilibrium dissociation constants (kd) for TAAs and CD3s after TCE (an IgG format, 150 kDa) administration once weekly at 1 mg/kg in patients.** Other parameters were the same as those for PF-06863135.
(DOCX)

S1 Code SBML code. (XML)
