# Supplementary material for: Leveraging a physiologically-based quantitative translational modeling platform for designing B cell maturation antigen-targeting bispecific T cell engagers for treatment of multiple myeloma
Source: PLoS Comput Biol. 2022 Jul 15;18(7):e1009715. doi: 10.1371/journal.pcbi.1009715 (PMC9328551; doi:10.1371/journal.pcbi.1009715)
Supplement: S1 Text — Table A. Summary of parameters related to the two-pore theory and systemic elimination of dAb2 and mAb in mice. Table B. Summary of physiological parameters in mice. Table C. Summary of baseline concentrations of tumor cells, T cells, and shed BCMAs in multiple myeloma patients. Fig A. In vitro TCE cytotoxicity model-simulated relationship between the average number of trimers per tumor cell and fractions viable or lysed of tumor cells of (A) PF-06863135 and (B) AMG420 in the absence or presence of shed BCMAs in in vitro cytotoxicity assays. Fig B. Schematic model simplification flow of the two-pore theory biodistribution model. (A) The original two-pore theory model explicitly accounted for a detailed tissue distribution process through perfusion, diffusion, and convection through two groups of pores (small and large pores) in a molecular size-dependent manner as well as uptake by vascular endothelial cells followed by endosomal degradation or an FcRn-mediated recycling mechanism in each tissue. (B) Model simplification process in which endosomal degradation and FcRn-mediated recycling mechanisms were removed and biodistribution rate constants in and out of tissue interstitial space (Q’ and (Q-L)’) were derived by integrating plasma flows as well as diffusion and convection rate constants via small and large pores under a quasi-steady state assumption. Tissue vascular spaces were lumped into central blood under a quasi-equilibrium assumption. (C) Simplified two-pore theory biodistribution model. The final model accounts for molecular size-dependent biodistribution between the central blood compartment and tissue interstitial spaces as well as lymphatic recirculation. Q, Q-L, L, PS, J, and Jiso represent arterial plasma flows, venous plasma flows, lymphatic flows, permeability surface areas, convectional flows, and isogravimetric lymph flows, respectively. Subscript BM, SP, LN, Tis, L, and S represent bone marrow, spleen, lymph node, other tissue, large pore, and sm [file pcbi.1009715.s001.docx]

**S1 Text.**

**Leveraging a physiologically-based quantitative translational modeling platform for designing B cell maturation antigen-targeting bispecific T cell engagers for treatment of multiple myeloma**

Tomoki Yoneyama,^1^ Mi-Sook Kim,^2^ Konstantin Piatkov, ^2^ Haiqing Wang^2^ and Andy Z.X. Zhu^1^

^1^Quantitative Solutions, Takeda Pharmaceuticals International Co., Cambridge, MA, USA.

^2^Global Drug Metabolism and Pharmacokinetics, Takeda Pharmaceuticals International Co., Cambridge, MA, USA.

# Supporting methods

## A bispecific TCE binding model for an in vitro cytotoxicity assay in the presence of shed targets

Bispecific T cell engagers (TCEs) redirect cytotoxic T cells to tumor associated antigens (TAAs), thereby forming immune synapses to exert anti-tumor effects. Bispecific TCE binding was characterized by a sequential mechanistic binding kinetics model. A schematic description of the TCE binding model structure is shown in Fig 1(A). TCEs bind to either TAAs or clusters of differentiation 3 (CD3s), depending on binding rate constants and target expression levels, to form TAA-TCE or TCE-CD3 dimers, followed by subsequent binding with another binding target of either CD3s or TAAs, respectively, to form TAA-TCE-CD3 trimers (immune synapses). Concentrations of TAAs or CD3s were calculated from cell numbers, TAA or CD3 expression levels per cell, and assay volumes. Shed targets competitively inhibit TCE binding to TAAs. In addition, multiple TCE species compete for CD3 binding, including TCEs, TAA-TCE dimers, or shed target-TCE dimers. Tumor growth was described by an exponential growth model depending on total TAA concentrations (the sum of TAAs, TAA-TCE dimers, and TAA-TCE-CD3 trimers). The tumor growth rate without a TCE treatment was estimated using the in vitro tumor growth data of L363 tumor reported in the literature [1]. Immune synapse-mediated tumor cell killing is a function of immune synapse and total TAA concentrations since immune synapse mediated tumor killing can decrease targets that do not form immune synapses but express on the tumor cells being killed by immune synapses. Differential equations are shown below.

$$\frac{dTCE}{dt}= -{kon}_{TAA}\cdot TCE\cdot TAA-{kon}_{CD3}\cdot TCE\cdot CD3-{kon}_{sTAA}\cdot TCE\cdot[shed target]+{koff}_{TAA}\cdot\left[ TAA-TCE \right]+{koff}_{CD3}\cdot\left[ TCE-CD3 \right]+{koff}_{sTAA}\cdot\left[ shed target-TCE \right] \cdots Eq S1$$

$$\frac{dTAA}{dt}=k_{g}\cdot(TAA+\left[ TAA-TCE \right]+[TAA-TCE-CD3])-{kon}_{TAA}\cdot TCE\cdot TAA-{kon}_{TAA}\cdot\left[ TCE-CD3 \right]\cdot TAA+{koff}_{TAA}\cdot\left[ TAA-TCE \right]+{koff}_{TAA}\cdot\left[ TAA-TCE-CD3 \right]-kkill\cdot TAA \cdots Eq S2$$

$$\frac{dCD3}{dt}=-{kon}_{CD3}\cdot TCE\cdot CD3-{kon}_{CD3}\cdot\left[ TAA-TCE \right]\cdot CD3-{kon}_{CD3}\cdot\left[ shed target-TCE \right]\cdot CD3+{koff}_{CD3}\cdot\left[ TCE-CD3 \right]+{koff}_{CD3}\cdot\left[ TAA-TCE-CD3 \right]+{koff}_{CD3}\cdot\left[ shed target-TCE-CD3 \right] \cdots Eq S3$$

$$\frac{d[shed target]}{dt}=-{kon}_{sTAA}\cdot TCE\cdot\left[ shed target \right]-{kon}_{sTAA}\cdot\left[ TCE-CD3 \right]\cdot[shed target]+{koff}_{sTAA}\cdot\left[ shed target-TCE \right]+{koff}_{sTAA}\cdot\left[ shed target-TCE-CD3 \right] \cdots Eq S4$$

$$\frac{d[TAA-TCE]}{dt}={kon}_{TAA}\cdot TCE\cdot TAA-{koff}_{TAA}\cdot\left[ TAA-TCE \right]-{kon}_{CD3}\cdot\left[ TAA-TCE \right]\cdot CD3+{koff}_{CD3}\cdot\left[ TAA-TCE-CD3 \right] -kkill\cdot[TAA-TCE] \cdots Eq S5$$

$$\frac{d[TCE-CD3]}{dt}={kon}_{CD3}\cdot TCE\cdot CD3-{koff}_{CD3}\cdot\left[ TCE-CD3 \right]-{kon}_{TAA}\cdot\left[ TCE-CD3 \right]\cdot TAA+{koff}_{TAA}\cdot\left[ TAA-TCE-CD3 \right]-{kon}_{sTAA}\cdot\left[ TCE-CD3 \right]\cdot sTAA+{koff}_{sTAA}\cdot\left[ shed target-TCE-CD3 \right] \cdots Eq S6$$

$$\frac{d[shed target-TCE]}{dt}={kon}_{sTAA}\cdot TCE\cdot[shed target]-{koff}_{sTAA}\cdot\left[ shed target-TCE \right]-{kon}_{CD3}\cdot\left[ shed target-TCE \right]\cdot CD3+{koff}_{CD3}\cdot\left[ shed target-TCE-CD3 \right] \cdots Eq S7$$

$$\frac{d[TAA-TCE-CD3]}{dt}={kon}_{CD3}\cdot\left[ TAA-TCE \right]\cdot CD3-{koff}_{CD3}\cdot\left[ TAA-TCE-CD3 \right]+{kon}_{TAA}\cdot\left[ TCE-CD3 \right]\cdot TAA+{koff}_{TAA}\cdot\left[ TAA-TCE-CD3 \right]-kkill\cdot[TAA-TCE-CD3] \cdots Eq S8$$

$$\frac{d[shed target -TCE-CD3]}{dt}={kon}_{CD3}\cdot\left[ shed target-TCE \right]\cdot CD3-{koff}_{CD3}\cdot\left[ shed target-TCE-CD3 \right]+{kon}_{sTAA}\cdot\left[ TCE-CD3 \right]\cdot[shed target]+{koff}_{sTAA}\cdot\left[ shed target-TCE-CD3 \right] \cdots Eq S9$$

$$\left[ trimer per tumor cell \right]=\frac{\left[ TAA-TCE-CD3 \right]}{TAA+\left[ TAA-TCE \right]+\left[ TAA-TCE-CD3 \right]}\cdot\left[ target per tumor cell \right]\cdots Eq S10$$

$$kkill=\frac{kii,max\cdot{[trimer per tumor cell]}^{hill}}{{kkill50}^{hill}+{[trimer per tumor cell]}^{hill}} \cdots Eq S11$$

$$\frac{d{TAA}_{control}}{dt}=k_{g}\cdot\left[ {TAA}_{control} \right] \cdots Eq S12$$

$$[viable tumor fraction]=\frac{TAA+\left[ TAA-TCE \right]+[TAA-TCE-CD3]}{{TAA}_{control}} \cdots Eq S13$$

Where TCE, TAA, [shed target], [TAA-TCE], [TCE-CD3], [shed target-TCE], [TAA-TCE-CD3], and [shed target-TCE-CD3] are concentrations of single species and their dimers and trimers. TAA_control_ is TAA concentration without a TCE treatment. kon_TAA_, kon_CD3_, and kon_sTAA_ as well as koff_TAA_, koff_CD3_, and koff_sTAA_ are association as well as dissociation rate constants of TCEs against TAAs, CD3s, and shed targets, respectively. Koff was derived from equilibrium dissociation rate constants (kd) and association rate constants (kon) under the equation of koff=kon*kd. [viable tumor fraction], kg, kkill,max, kkill50, hill, and [target per tumor cell] represent a fraction of viable tumor cells, a tumor growth rate, a maximum tumor kill rate by immune synapses, the half-maximum number of immune synapses per tumor cell, a hill coefficient associated with a tumor kill rate by immune synapses, and target expression per tumor cell, respectively.

## Simplification of a molecular size-dependent two-pore theory biodistribution model for different sizes of proteins in mice

The biodistribution of different molecular sizes of TCEs and shed targets was delineated using the two-pore theory biodistribution model adapted from the literature [2]. A schematic description of the two-pore theory biodistribution model structure is shown in Fig 1(B). While sixteen of the major tissues were included in the original model, our model focused on bone marrow as a tumor site of multiple myeloma as well as spleen and lymph nodes as lymphatic organs. Tissues were connected by blood circulation through arterial plasma flow (Q) and venous plasma flow (Q-L). Lymphatic flows (L) generated from vascular spaces to interstitial spaces flow into lymph node and eventually recirculate into venous plasma. Molecular size-dependent tissue disposition of proteins has been characterized by the two-pore theory, in which proteins are transported from vascular spaces to interstitial spaces via two groups of pores (small pores: ~4.44 nm, large pores: ~22.9 nm) by diffusion and convection. Equations to determine molecular size-dependent transport processes under the two-pore theory are as follows;

$${PS}_{L,j}= \frac{L_{j}\cdot{Xp}_{L}\cdot{Pe}_{L,j}}{e^{{Pe}_{L,j}}-1} \cdots Eq S11$$

$${PS}_{S,j}= \frac{L_{j}\cdot{Xp}_{S}\cdot{Pe}_{S,j}}{e^{{Pe}_{S},j}-1} \cdots Eq S12$$

$$J_{L,j}= L_{j}\cdot\left( \alpha_{L}+xj \right)\cdot\left( 1-\sigma_{v,L} \right) \cdots Eq S13$$

$$J_{S,j}= L_{j}\cdot\left( \alpha_{S}-xj \right)\cdot\left( 1-\sigma_{v,S} \right) \cdots Eq S14$$

$${Pe}_{L,j}=\frac{L_{j}\cdot\left\{ \left( \alpha_{L}+xj \right)\cdot\left( 1-\sigma_{v,L} \right) \right\}}{L_{j}\cdot{Xp}_{L}} \cdots Eq S15$$

$${Pe}_{S,j}=\frac{L_{j}\cdot\left\{ \left( \alpha_{S}+xj \right)\cdot\left( 1-\sigma_{v,S} \right) \right\}}{L_{j}\cdot{Xp}_{S}} \cdots Eq S16$$

$${Xp}_{L}=\frac{RT}{6\pi N}\cdot\frac{8}{\Delta P-\overline{\sigma_{a}}\cdot\Delta\pi}\cdot\frac{1}{a_{e}}\cdot\frac{A_{L}}{A_{0,L}}\cdot\frac{\alpha_{L}}{r_{L}^{2}} \cdots Eq S17$$

$${Xp}_{S}=\frac{RT}{6\pi N}\cdot\frac{8}{\Delta P-\overline{\sigma_{a}}\cdot\Delta\pi}\cdot\frac{1}{a_{e}}\cdot\frac{A_{S}}{A_{0,S}}\cdot\frac{\alpha_{S}}{r_{S}^{2}} \cdots Eq S18$$

$$a_{e}=0.0483\cdot{MW}^{0.386} \cdots Eq S19$$

$$\sigma_{v,L}=0.000035\cdot{MW}^{0.717} \cdots Eq S20$$

$$\sigma_{v,S}=1-0.8489\cdot e^{-0.00004\cdot MW} \cdots Eq S21$$

$$\frac{A_{L}}{A_{0,L}}=0.3429\cdot e^{-0.00012175\cdot MW}+0.6571\cdot e^{-0.00000421\cdot MW} \cdots Eq S22$$

$$\frac{A_{S}}{A_{0,S}}=0.2352\cdot e^{-0.0008295\cdot MW}+0.7767\cdot e^{-0.00053095\cdot MW} \cdots Eq S23$$

Where PS, J, and L represent permeability surface areas, convectional flows, and lymphatic flows, respectively. Subscript L, S, and j represent large pore, small pore, and jth tissue, respectively. Pe and σ_v_ represent the Peclet number and a vascular reflection coefficient, respectively. Xp is a constant linking PS to L, depending on pore sizes, relative hydraulic conductances of large and small pores, and protein sizes. xj is a constant linking isogravimetric lymph flow to L. a_e_, r, α, A/A_0_, R, T, N, Δ𝑃−$\overline{\sigma_{a}}$∙Δ𝜋, and MW represent the Stokes-Einstein radius, pore sizes, fractional hydraulic conductance of pores, fractional accessible pore sizes, a gas constant, the body temperature, the Avogadro constant, a Starling force, and molecular weight in Dalton, respectively.

The original full-tissue two-pore model was simplified, and a schematic model simplification flow is depicted in S2 Fig. Model simplification took the following four steps: 1) tissues except for bone marrow, spleen, and lymph node were lumped into one other tissue compartment; 2) endosomal compartments in tissues and their associated degradation of proteins were removed, and endosomal degradation of proteins was accounted for by estimating systemic clearance; 3) tissue vascular spaces were lumped into one central blood compartment assuming quasi-equilibrium; and 4) biodistribution processes between central blood and tissue interstitial spaces through vascular spaces were further simplified under quasi-steady state assumption. The simplified distribution rate constants in-between central blood and interstitial spaces (Q’ and (Q-L)’) were derived by integrating perfusion as well as diffusion and convectional rate constants through small and large pores in each compartment, as shown in Eqs 1 and 2 in Materials and Methods section. Tissue concentrations in the bone marrow and spleen were reconstituted by the equation below.

$$C_{tot, BM or SP}=\frac{C_{PL}\cdot V_{vas, BM or SP}+C_{IS, BM or SP}\cdot V_{IS, BM or SP}}{V_{tot, BM or SP}} \cdots Eq S24$$

Where C_tot_, C_PL_, and C_IS_ represent concentrations in total tissues, plasma, and interstitial spaces, respectively. V_tot_, V_vas_, and V_IS_ represent volumes of total tissues, vascular spaces, and interstitial spaces, respectively. BM and SP represent bone marrow and spleen, respectively.

## Translation and verification of a two-pore biodistribution model for TCEs in patients

The simplified and verified two-pore biodistribution model in mice was translationally scaled into humans by replacing the physiological parameters including blood and lymphatic flow rates as well as blood and tissue volumes. The lymphatic flow rates in bone marrow and spleen in humans were derived by multiplying the lymphatic flow rate ratios in mice between Sepp et al [3] and Shah et al [4] to human lymphatic flow rates reported by Shah et al [4]. The model was further integrated with a T cell biodistribution model adapted from literature [5] with similar simplification of transmigration of T cells from central blood to tissue interstitial spaces via tissue vascular spaces.

$${Q''}_{j}= \frac{Q_{j}\cdot T_{j}}{Q_{j}-L_{j}+T_{j}} \cdots Eq S25$$

Where T_J_ represents a transmigration rate of T cells in jth tissue. The integrated transmigration rates were applied to tumor cells as well. The baseline concentrations of T cells and tumor cells in blood and tissues in multiple myeloma patients were calculated using values collected from literature [6-11]. Multiple myeloma cells in bone marrow were assumed to be 20% of total cells. T cell progenitors are generated in bone marrow, matured in thymus, and then degraded in liver and spleen. Therefore, in our model, T cells were assumed to be synthesized with a zero order synthesis rate in other tissue compartment and degraded in spleen and other tissue compartment with a first order elimination rate [12]. Turnover of tumor cells was not considered. Under these assumptions, tissue retention factors of T cells and tumor cells were calculated under the steady state condition. Finally, the bispecific binding model was incorporated into all the tissues, central blood, and lymph node compartments.

The first order elimination rate of shed BCMAs in patients was estimated from molecular weight of shed BCMA (5000 Da) using the empirical equation below [13].

$$k_{deg,sBCMA}=e^{1-\frac{7.47}{{1+e}^{0.003618\cdot(23.24-MW)}}}\cdot\left( \frac{{BW}_{human}}{{BW}_{mice}} \right)^{0.75}/Vp \cdots Eq 26$$

Where MW, BW, and Vp represent molecular weight, body weight, and plasma volume, respectively. A zero order synthesis rate of shed BCMAs in bone marrow was estimated under quasi-steady state assumption to sustain observed blood concentration (100 nM). Likewise, the baseline shed BCMA concentrations in tissue compartments were estimated by the two-pore biodistribution model. A summary of baseline concentrations of tumor cells, T cells, and shed BCMAs is shown in S3 Table.

Table A. Summary of parameters related to the two-pore theory and systemic elimination of dAb_2_ and mAb in mice

| Parameter | Unit | Description | Value (%RSE) | | Source |
| --- | --- | --- | --- | --- | --- |
|  |  |  | dAb_2_ | mAb |  |
| MW | Da | molecular weight | 25600 | 150000 | [3, 4] |
| a_e_ | nm | Stokes-Einstein radius | 2.43 | 4.81 | calc by MW |
| σ_vL_ | - | vascular reflection coefficient of large/small pore | 0.0507 | 0.18 | calc by MW |
| σ_vS_ | - | vascular reflection coefficient of large/small pore | 0.695 | 0.998 | calc by MW |
| A_L_/A_0L_ | - | fractional accessible large pore size | 0.605 | 0.349 | calc by MW |
| A_S_/A_0S_ | - | fractional accessible small pore size | 0.0281 | 9.28E-07 | calc by MW |
| r_L_ | nm | large pore size | 22.85 | | [2] |
| r_S_ | - | small pore size | 4.44 | | [2] |
| α_L_ | - | fractional hydraulic conductance of large pore | 0.024 | | [2] |
| α_S_ | - | fractional hydraulic conductance of small pore | 0.958 | | [2] |
| xj | - | coefficient for isogravimetric lymph flow | 0.38 | | [2] |
| Δ𝑃−$\overline{\sigma_{a}}$∙Δ𝜋 | mmHg | starling force | 1 | | [2] |
| CL | L/h/kg | plasma clearance | 0.254 (6.9) | 0.000600 (20.2) | estimated |
| Proportional error |  |  | 0.39 | 0.165 |  |

Table B. Summary of physiological parameters in mice

| Tissue | Total volume (L/kg) | Plasma volume (L/kg) | Interstitial volume (L/kg) | Plasma flow (L/h/kg) | Lymphatic flow (L/h/kg) | | Lymphatic reflection coefficient |
| --- | --- | --- | --- | --- | --- | --- | --- |
|  |  |  |  |  | Sepp et al | Shah et al |  |
| plasma | 0.0337 | - | - | 13.32 | - | - | - |
| bone marrow | 0.101 | 0.00222 | 0.0188 | 0.54 | 0.010 | 0.0011 | 0.2 |
| spleen | 0.00454 | 0.00055 | 0.000907 | 0.29 | 0.000024 | 0.0006 | 0.2 |
| other tissue | 0.833 | 0.0264 | 0.156 | 12.49 | 0.0516 | 0.0516 | 0.2 |
| lymph node | 0.00404 | - | - | - | 0.0616 | 0.0533 | - |
| Source | [4] | [4] | [4] | [4] | [3] | [4] | [4] |

Table C. Summary of baseline concentrations of tumor cells, T cells and shed BCMAs in multiple myeloma patients

| Tissue | E:T ratio | Tumor cell conc (nM) | T cell conc (nM) | BCMA (nM) | CD3 (nM) | shed BCMA (nM) |
| --- | --- | --- | --- | --- | --- | --- |
| plasma | 1016 | 3.87E-09 | 3.93E-06 | 0.0000487 | 0.393 | 100 |
| bone marrow | 0.555 | 6.59E-05 | 3.66E-05 | 0.830 | 3.66 | 345* |
| spleen | 354 | 2.77E-06 | 9.83E-04 | 0.0349 | 98.26 | 100* |
| other tissue | 728 | 3.03E-08 | 2.21E-05 | 0.000382 | 2.21 | 100* |
| lymph node | 552 | 1.17E-06 | 6.45E-04 | 0.0147 | 64.5 | 91.9* |
| Source | calculated | [6, 10, 11] | [6-9] | calculated | calculated | [14]  *calculated by the model |

BCMA expression per tumor cell: 12590 [15], CD3 expression per T cell: 100000 [16]


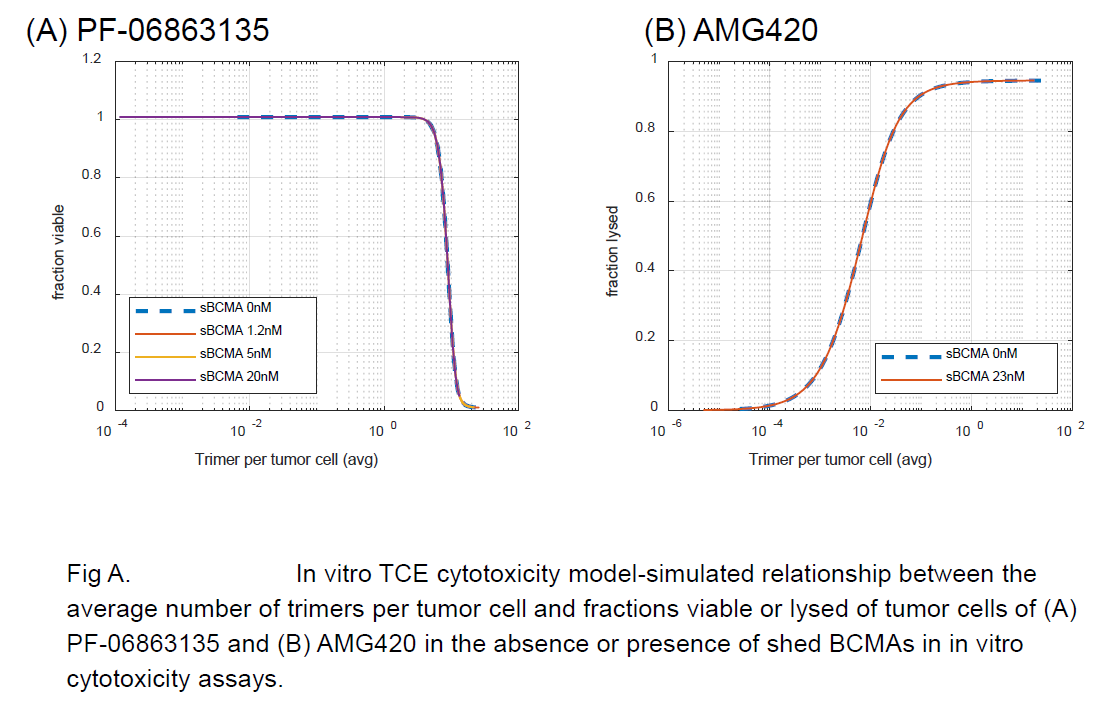


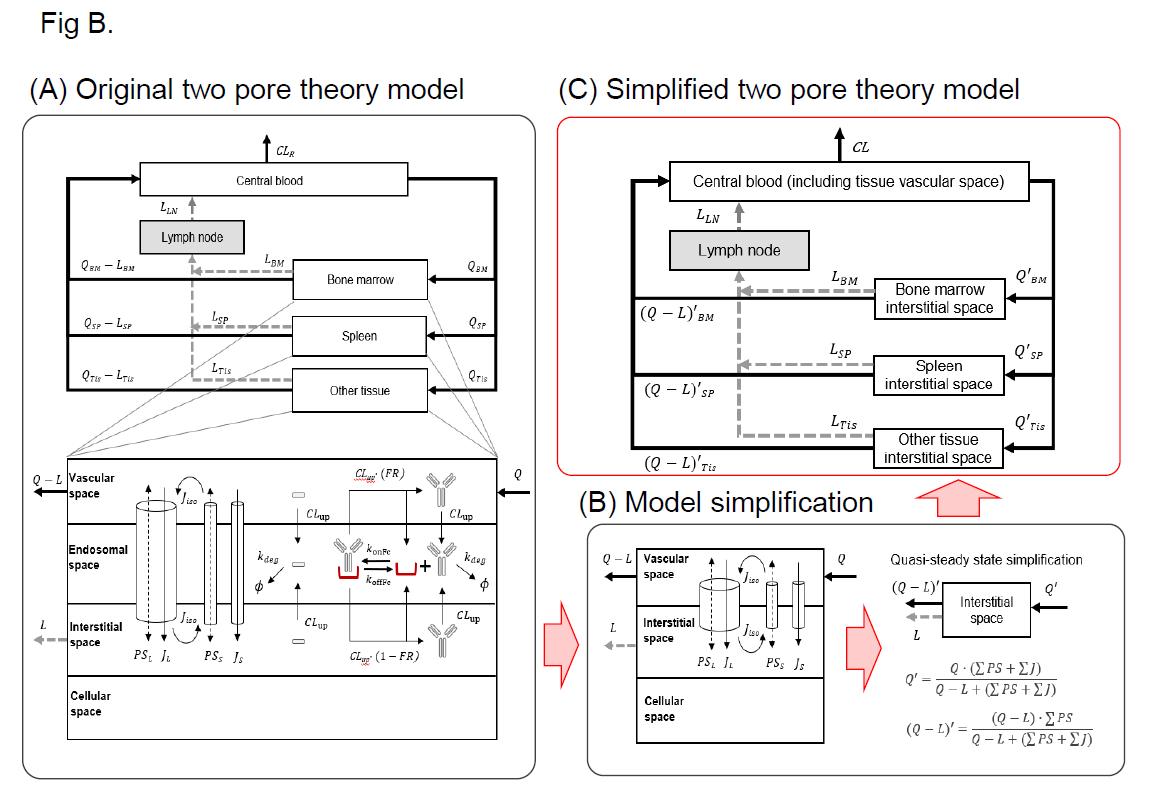


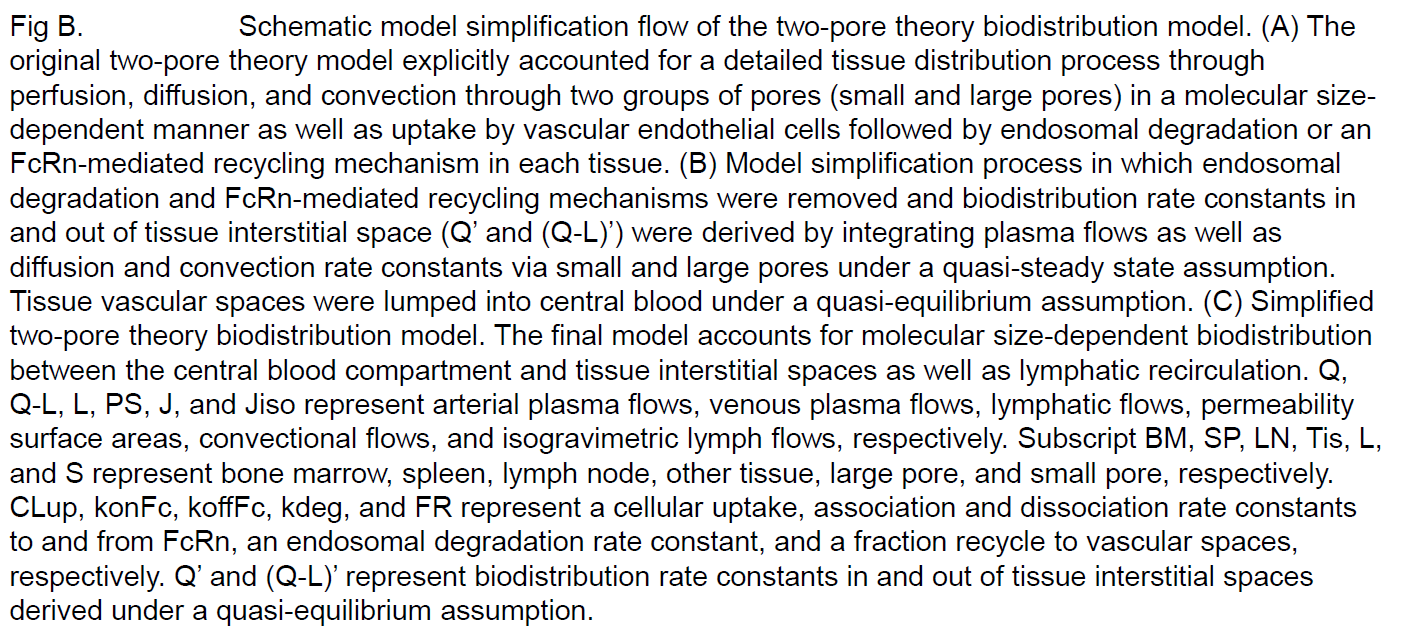


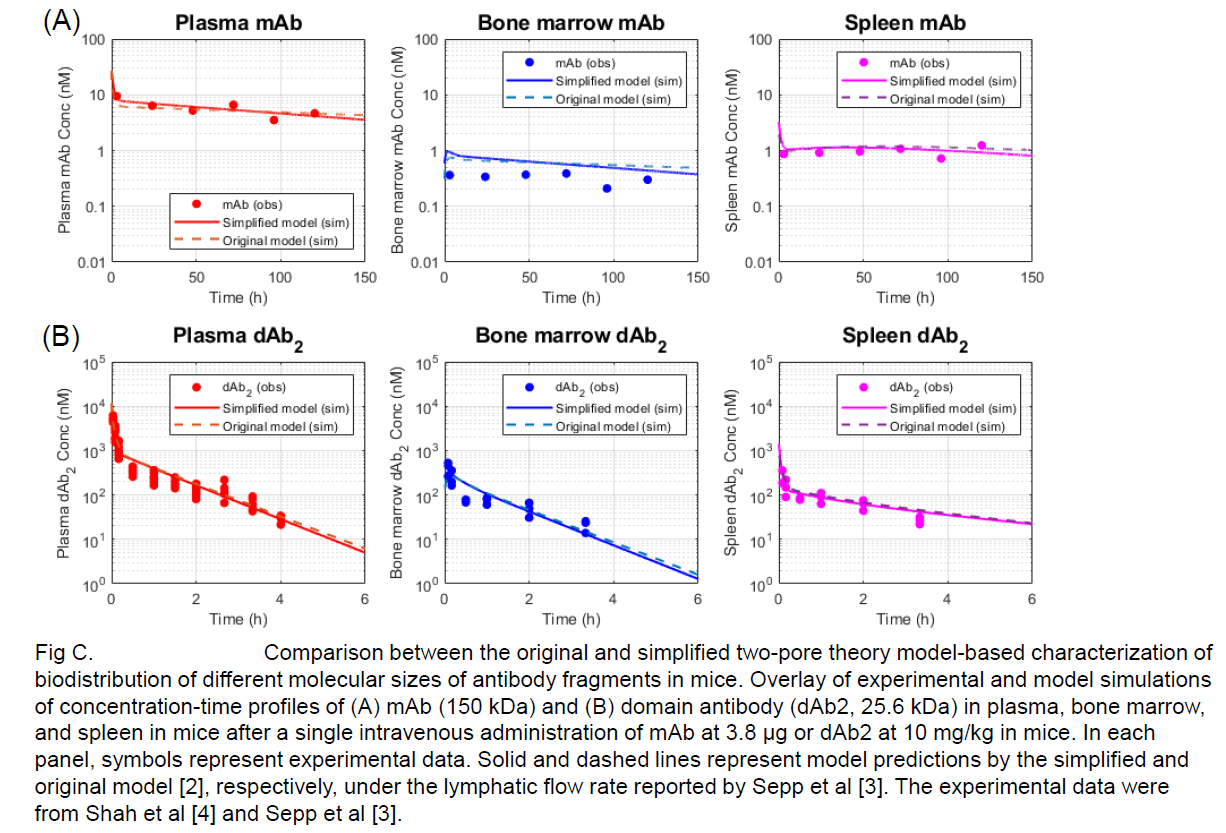


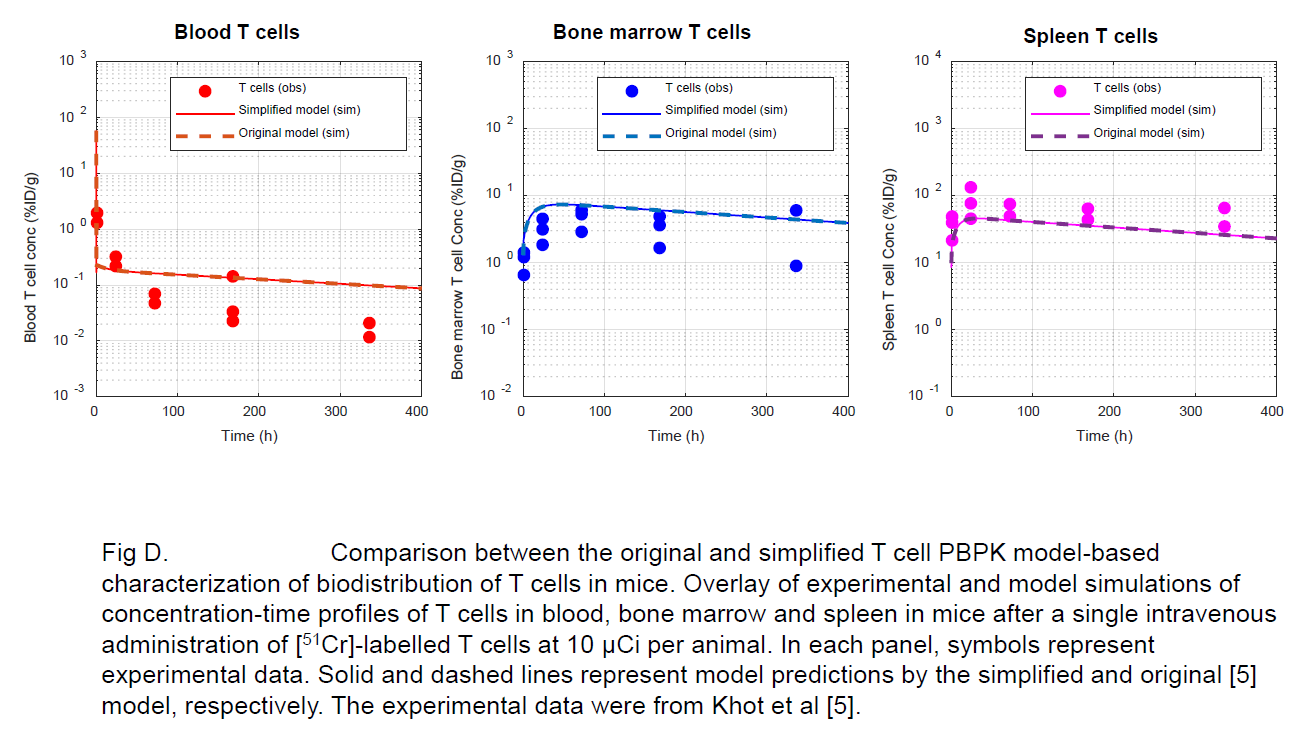


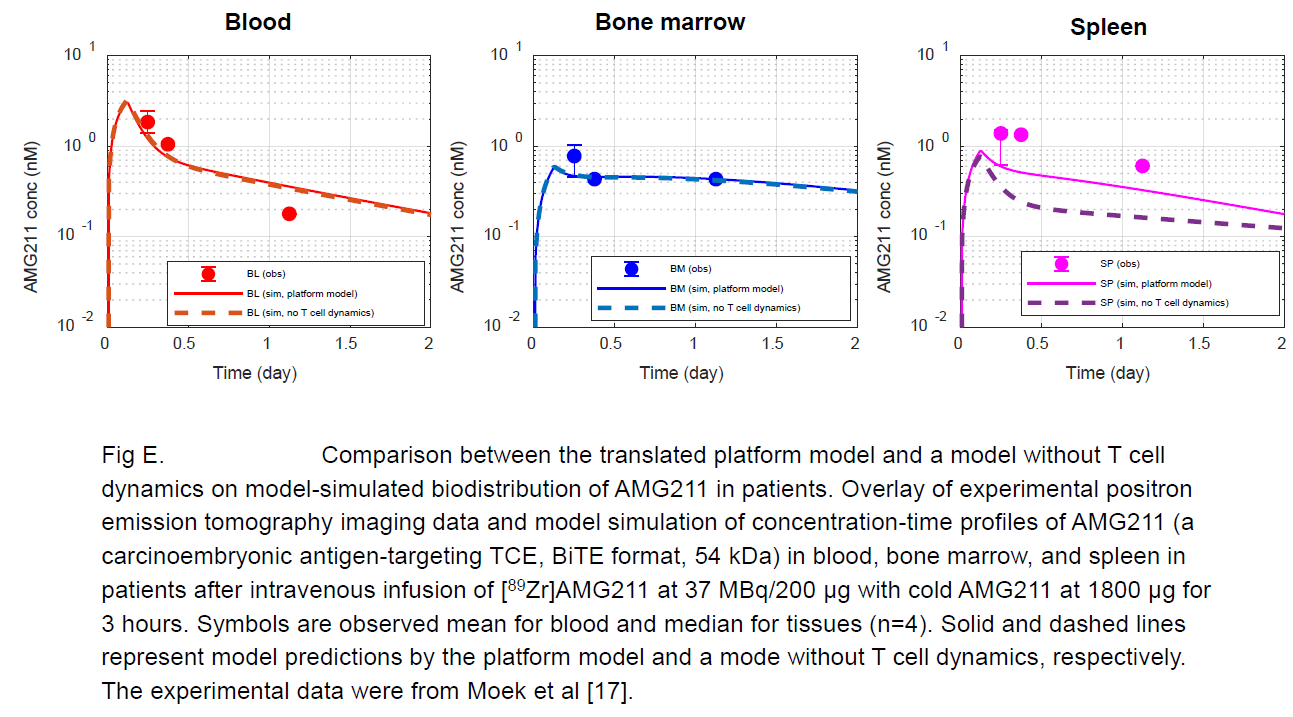


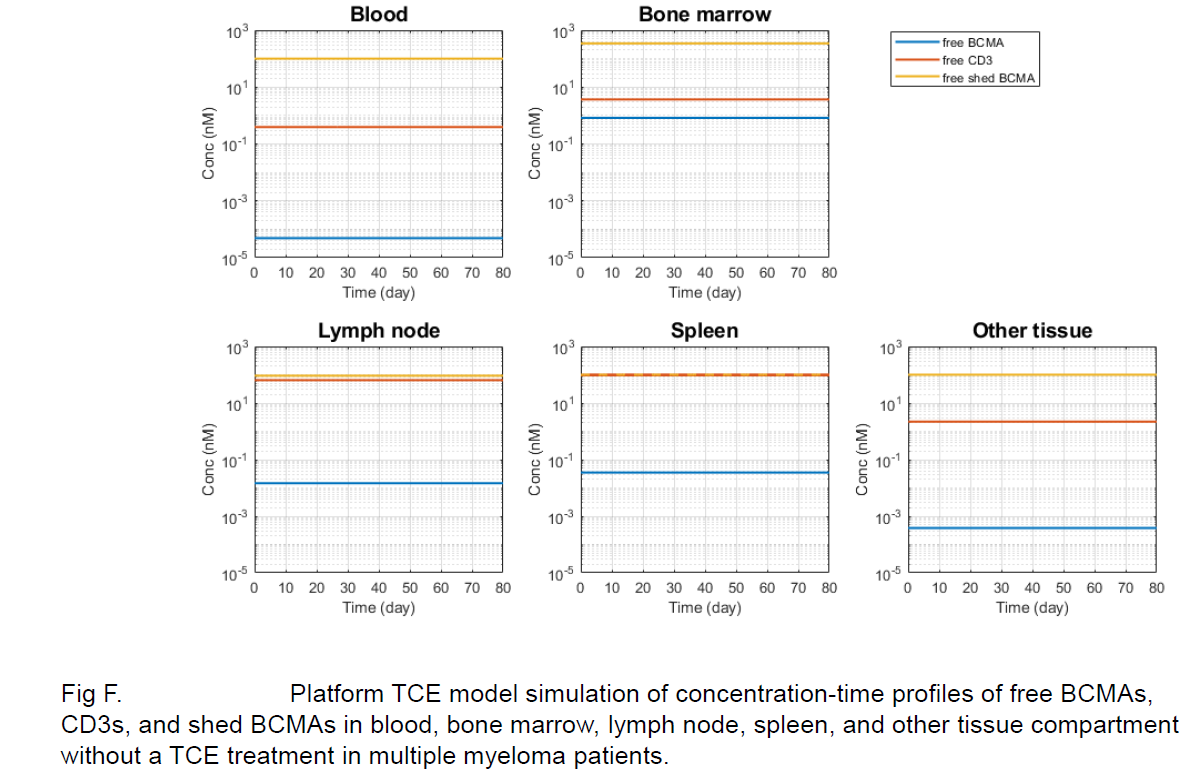


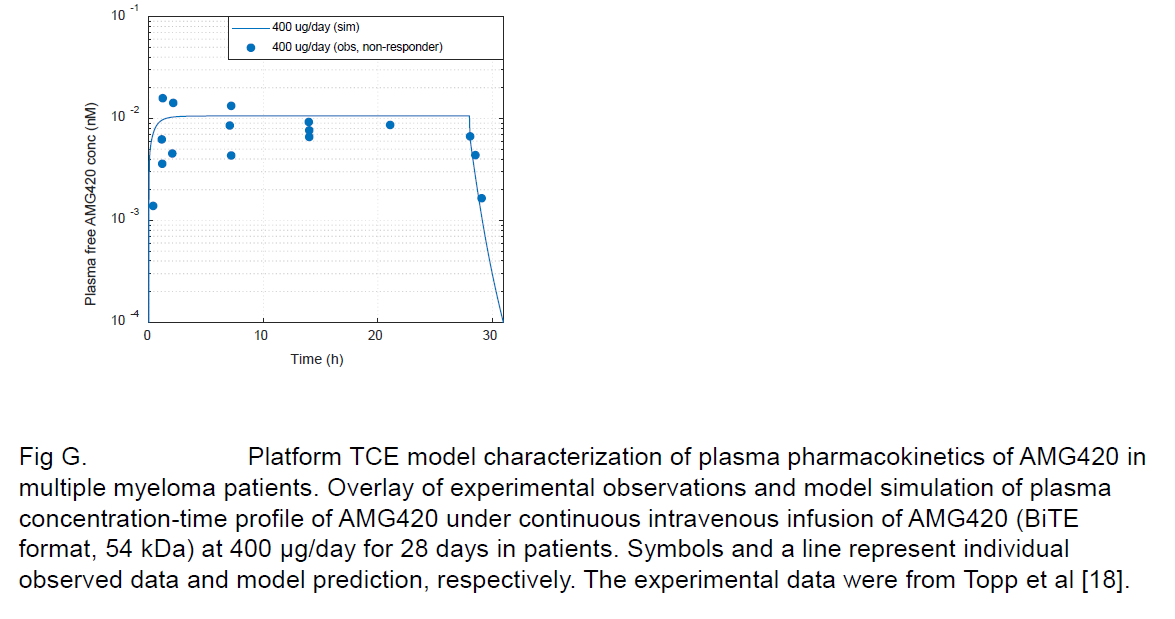


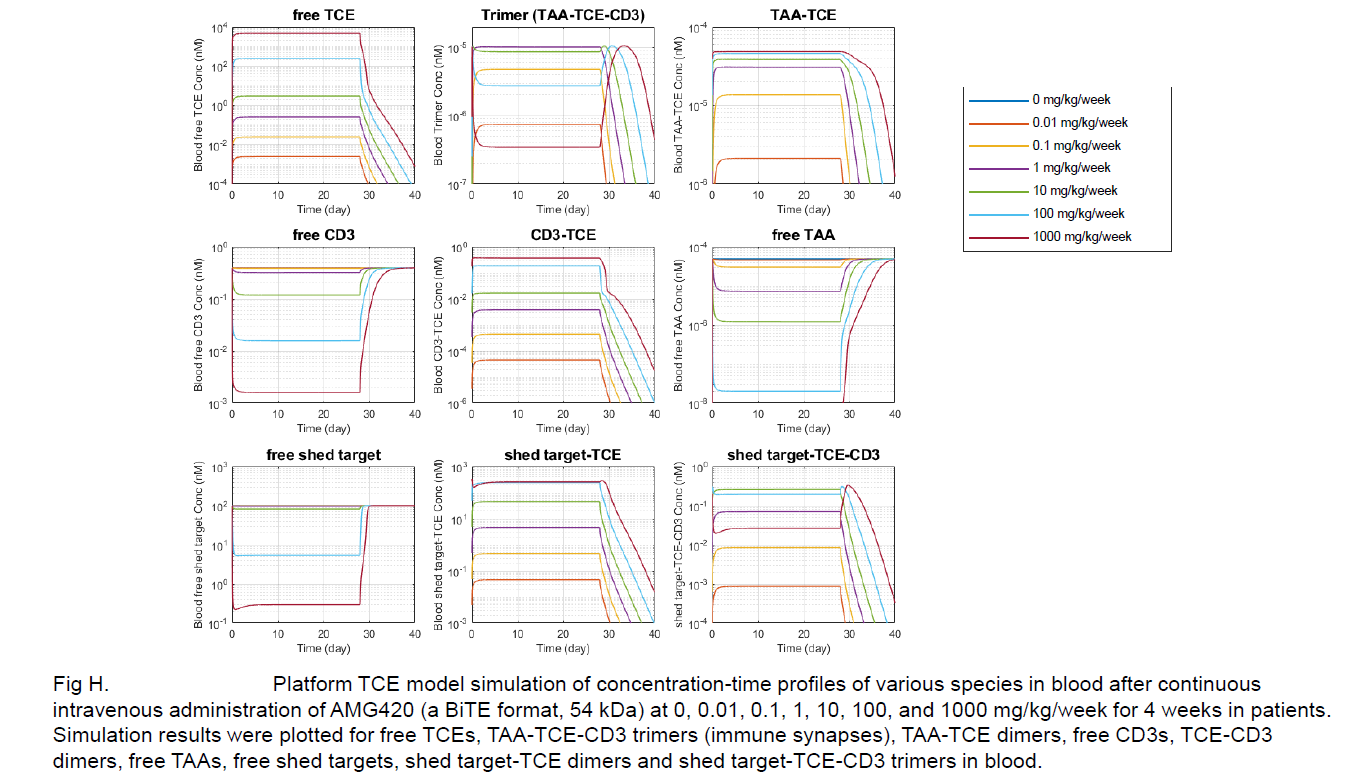


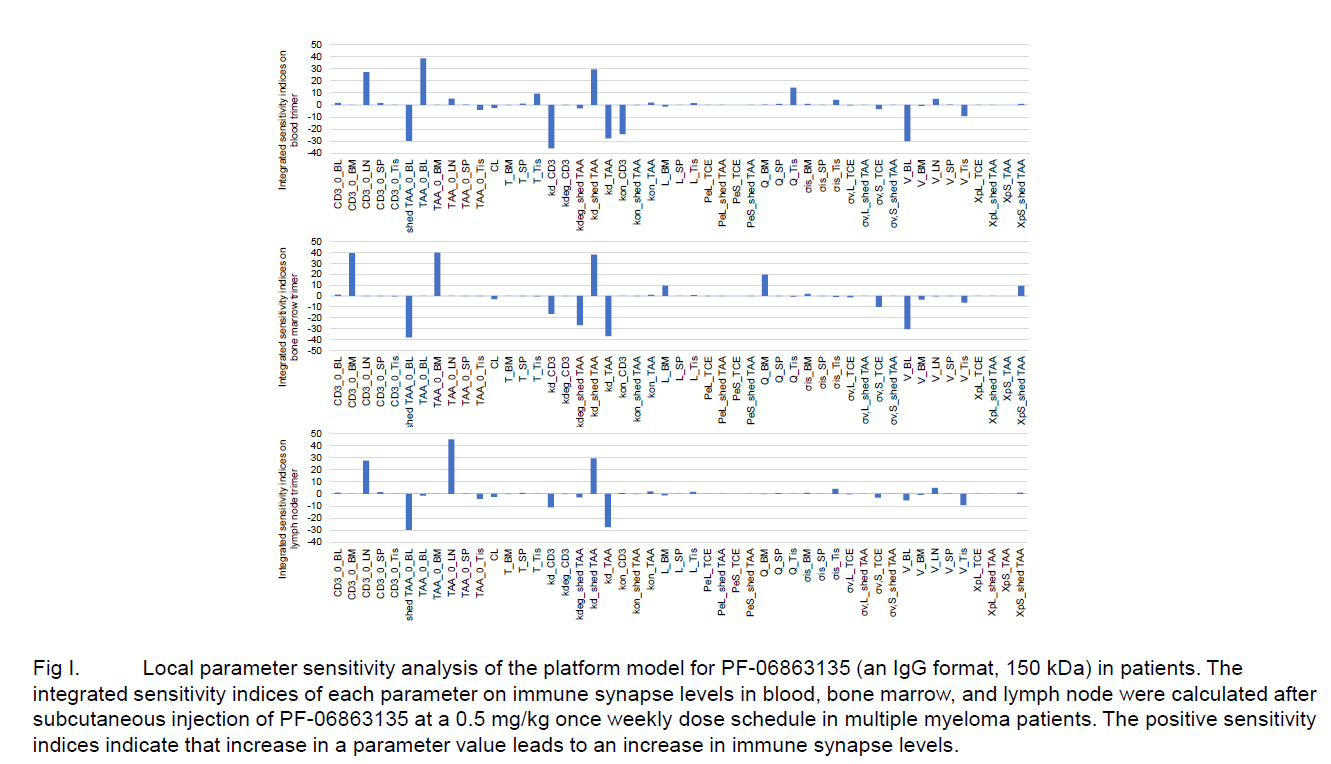


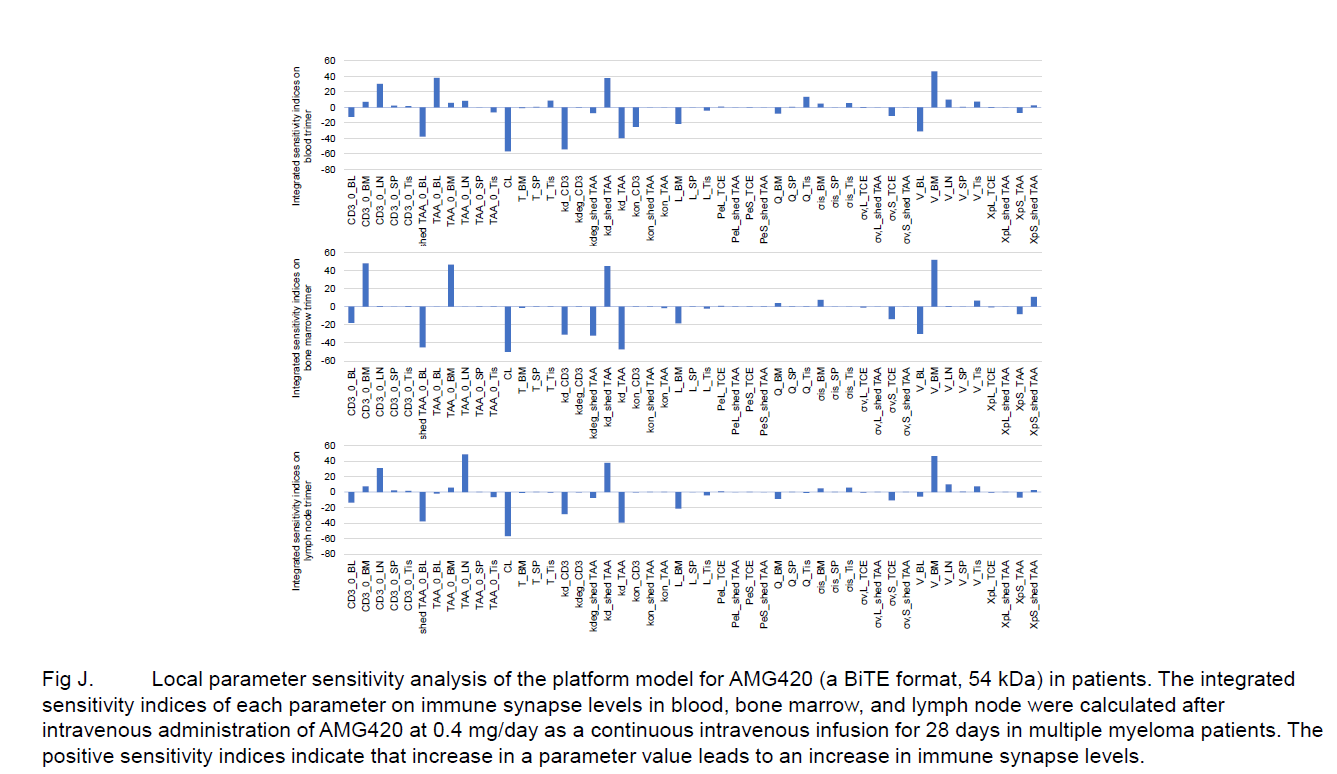


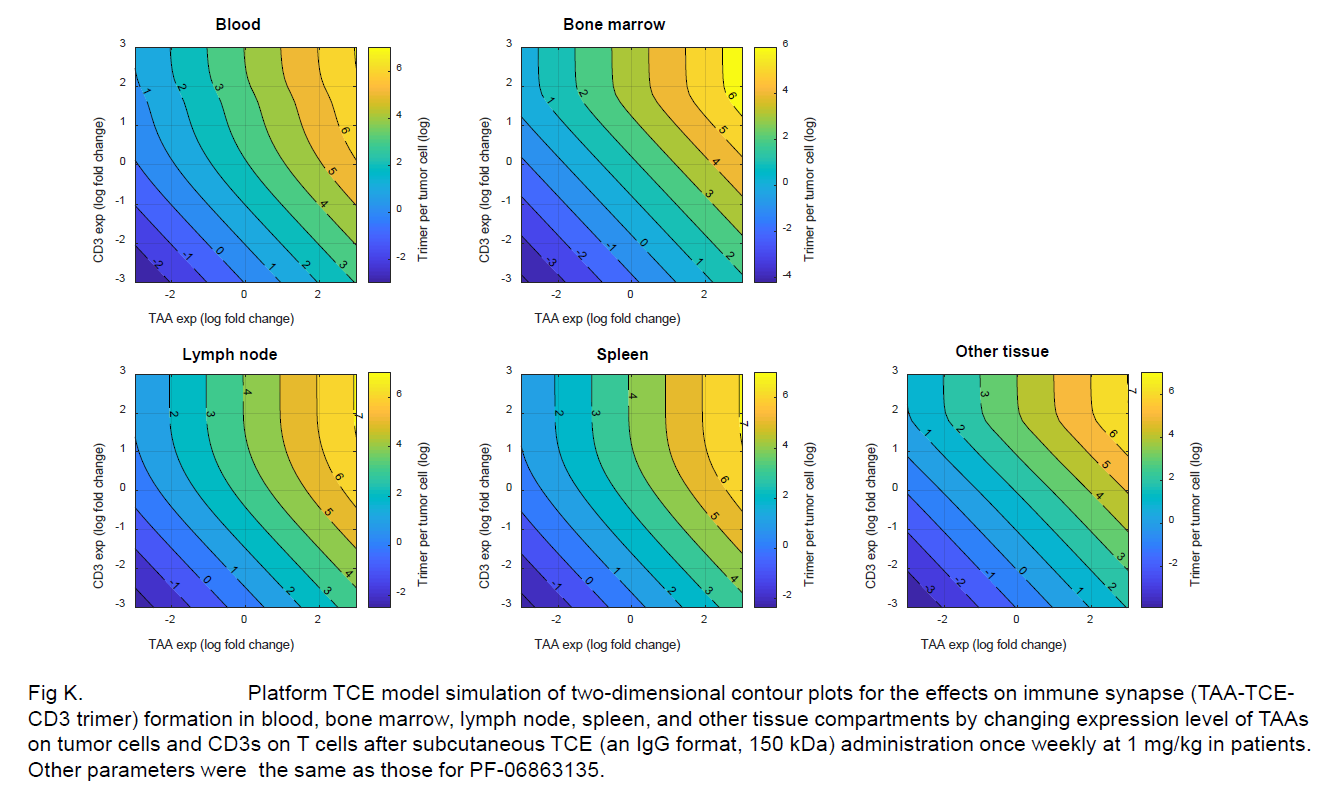


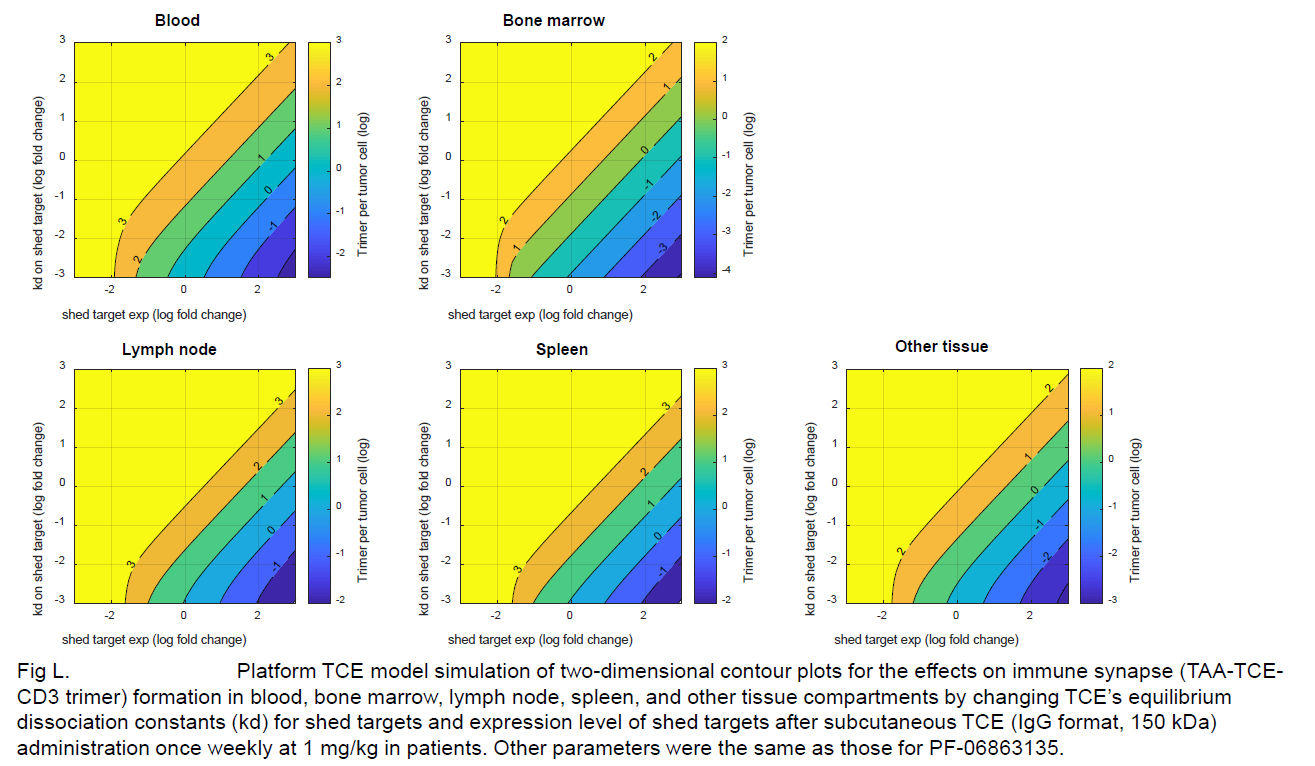


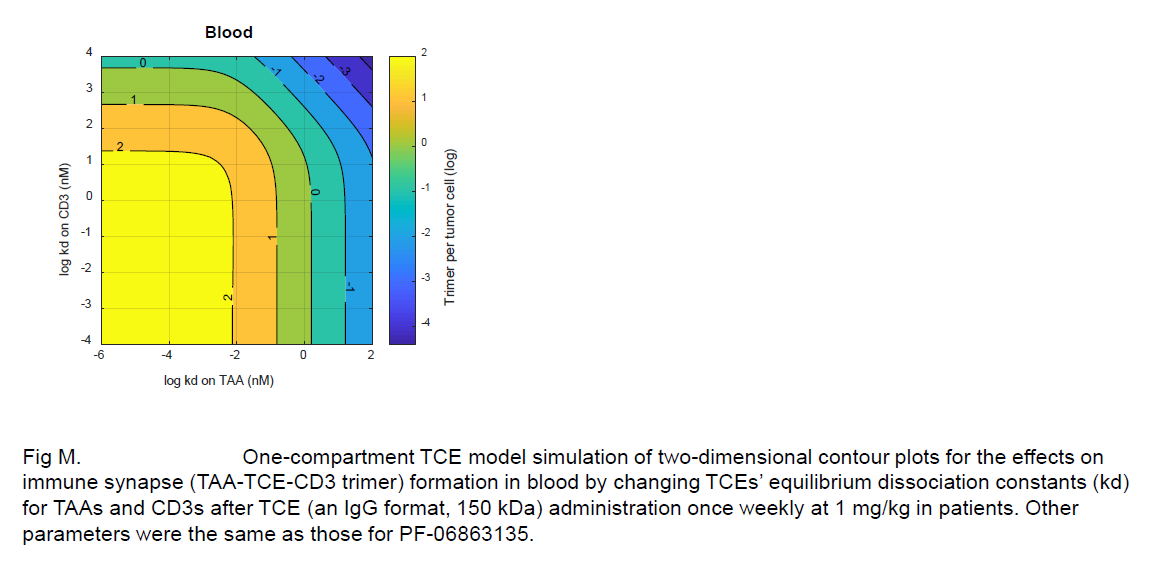


# References

1. Zlei M, Egert S, Wider D, Ihorst G, Wasch R, Engelhardt M. Characterization of in vitro growth of multiple myeloma cells. Exp Hematol. 2007;35(10):1550-61. Epub 2007/09/25. doi: 10.1016/j.exphem.2007.06.016. PubMed PMID: 17889722.

2. Li Z, Shah DK. Two-pore physiologically based pharmacokinetic model with de novo derived parameters for predicting plasma PK of different size protein therapeutics. J Pharmacokinet Pharmacodyn. 2019;46(3):305-18. Epub 2019/04/28. doi: 10.1007/s10928-019-09639-2. PubMed PMID: 31028591; PubMed Central PMCID: PMCPMC6531344.

3. Sepp A, Berges A, Sanderson A, Meno-Tetang G. Development of a physiologically based pharmacokinetic model for a domain antibody in mice using the two-pore theory. J Pharmacokinet Pharmacodyn. 2015;42(2):97-109. Epub 2015/01/13. doi: 10.1007/s10928-014-9402-0. PubMed PMID: 25577033.

4. Shah DK, Betts AM. Towards a platform PBPK model to characterize the plasma and tissue disposition of monoclonal antibodies in preclinical species and human. J Pharmacokinet Pharmacodyn. 2012;39(1):67-86. Epub 2011/12/07. doi: 10.1007/s10928-011-9232-2. PubMed PMID: 22143261.

5. Khot A, Matsueda S, Thomas VA, Koya RC, Shah DK. Measurement and Quantitative Characterization of Whole-Body Pharmacokinetics of Exogenously Administered T Cells in Mice. J Pharmacol Exp Ther. 2019;368(3):503-13. Epub 2019/01/10. doi: 10.1124/jpet.118.252858. PubMed PMID: 30622170; PubMed Central PMCID: PMCPMC6382992.

6. Westermann J, Pabst R. Distribution of lymphocyte subsets and natural killer cells in the human body. Clin Investig. 1992;70(7):539-44. Epub 1992/07/01. doi: 10.1007/BF00184787. PubMed PMID: 1392422.

7. Battaglia A, Ferrandina G, Buzzonetti A, Malinconico P, Legge F, Salutari V, et al. Lymphocyte populations in human lymph nodes. Alterations in CD4+ CD25+ T regulatory cell phenotype and T-cell receptor Vbeta repertoire. Immunology. 2003;110(3):304-12. Epub 2003/11/25. doi: 10.1046/j.1365-2567.2003.01742.x. PubMed PMID: 14632657; PubMed Central PMCID: PMCPMC1783055.

8. Zhao E, Xu H, Wang L, Kryczek I, Wu K, Hu Y, et al. Bone marrow and the control of immunity. Cell Mol Immunol. 2012;9(1):11-9. Epub 2011/10/25. doi: 10.1038/cmi.2011.47. PubMed PMID: 22020068; PubMed Central PMCID: PMCPMC3251706.

9. Aggarwal N, Fischer J, Swerdlow SH, Craig FE. Splenic lymphoid subsets with less well-recognized phenotypes mimic aberrant antigen expression. Am J Clin Pathol. 2013;140(6):787-94. Epub 2013/11/15. doi: 10.1309/AJCPPIBH3I1VRWXQ. PubMed PMID: 24225744.

10. Greer JP, Arber DA, Glader BE, List AF, Means RT, Rodgers GM. Wintrobe's Clinical Hematology 14th edition. Wolters Kluwer. 2018.

11. Rawstron AC, Owen RG, Davies FE, Johnson RJ, Jones RA, Richards SJ, et al. Circulating plasma cells in multiple myeloma: characterization and correlation with disease stage. Br J Haematol. 1997;97(1):46-55. Epub 1997/04/01. doi: 10.1046/j.1365-2141.1997.72653.x. PubMed PMID: 9136941.

12. Qomlaqi M, Bahrami F, Ajami M, Hajati J. An extended mathematical model of tumor growth and its interaction with the immune system, to be used for developing an optimized immunotherapy treatment protocol. Math Biosci. 2017;292:1-9. Epub 2017/07/18. doi: 10.1016/j.mbs.2017.07.006. PubMed PMID: 28713023.

13. Li Z, Krippendorff BF, Shah DK. Influence of Molecular size on the clearance of antibody fragments. Pharm Res. 2017;34(10):2131-41. Epub 2017/07/07. doi: 10.1007/s11095-017-2219-y. PubMed PMID: 28681164; PubMed Central PMCID: PMCPMC5693627.

14. Ghermezi M, Li M, Vardanyan S, Harutyunyan NM, Gottlieb J, Berenson A, et al. Serum B-cell maturation antigen: a novel biomarker to predict outcomes for multiple myeloma patients. Haematologica. 2017;102(4):785-95. Epub 2016/12/31. doi: 10.3324/haematol.2016.150896. PubMed PMID: 28034989; PubMed Central PMCID: PMCPMC5395119.

15. Friedman KM, Garrett TE, Evans JW, Horton HM, Latimer HJ, Seidel SL, et al. Effective Targeting of Multiple B-Cell Maturation Antigen-Expressing Hematological Malignances by Anti-B-Cell Maturation Antigen Chimeric Antigen Receptor T Cells. Hum Gene Ther. 2018;29(5):585-601. Epub 2018/04/12. doi: 10.1089/hum.2018.001. PubMed PMID: 29641319; PubMed Central PMCID: PMCPMC5930946.

16. Chen X, Haddish-Berhane N, Moore P, Clark T, Yang Y, Li H, et al. Mechanistic Projection of First-in-Human Dose for Bispecific Immunomodulatory P-Cadherin LP-DART: An Integrated PK/PD Modeling Approach. Clin Pharmacol Ther. 2016;100(3):232-41. Epub 2016/05/14. doi: 10.1002/cpt.393. PubMed PMID: 27170541.

17. Moek KL, Waaijer SJH, Kok IC, Suurs FV, Brouwers AH, Menke-van der Houven van Oordt CW, et al. (89)Zr-labeled Bispecific T-cell Engager AMG 211 PET Shows AMG 211 Accumulation in CD3-rich Tissues and Clear, Heterogeneous Tumor Uptake. Clin Cancer Res. 2019;25(12):3517-27. Epub 2019/02/13. doi: 10.1158/1078-0432.CCR-18-2918. PubMed PMID: 30745297.

18. Topp MS, Duell J, Zugmaier G, Attal M, Moreau P, Langer C, et al. Anti-B-Cell Maturation Antigen BiTE Molecule AMG 420 Induces Responses in Multiple Myeloma. J Clin Oncol. 2020;38(8):775-83. Epub 2020/01/03. doi: 10.1200/JCO.19.02657. PubMed PMID: 31895611.

**Fig A. In vitro TCE cytotoxicity model-simulated relationship between the average number of trimers per tumor cell and fractions viable or lysed of tumor cells of (A) PF-06863135 and (B) AMG420 in the absence or presence of shed BCMAs in in vitro cytotoxicity assays.**

**Fig B. Schematic model simplification flow of the two-pore theory biodistribution model.** (A) The original two-pore theory model explicitly accounted for a detailed tissue distribution process through perfusion, diffusion, and convection through two groups of pores (small and large pores) in a molecular size-dependent manner as well as uptake by vascular endothelial cells followed by endosomal degradation or an FcRn-mediated recycling mechanism in each tissue. (B) Model simplification process in which endosomal degradation and FcRn-mediated recycling mechanisms were removed and biodistribution rate constants in and out of tissue interstitial space (Q’ and (Q-L)’) were derived by integrating plasma flows as well as diffusion and convection rate constants via small and large pores under a quasi-steady state assumption. Tissue vascular spaces were lumped into central blood under a quasi-equilibrium assumption. (C) Simplified two-pore theory biodistribution model. The final model accounts for molecular size-dependent biodistribution between the central blood compartment and tissue interstitial spaces as well as lymphatic recirculation. Q, Q-L, L, PS, J, and Jiso represent arterial plasma flows, venous plasma flows, lymphatic flows, permeability surface areas, convectional flows, and isogravimetric lymph flows, respectively. Subscript BM, SP, LN, Tis, L, and S represent bone marrow, spleen, lymph node, other tissue, large pore, and small pore, respectively. CLup, konFc, koffFc, kdeg, and FR represent a cellular uptake, association and dissociation rate constants to and from FcRn, an endosomal degradation rate constant, and a fraction recycle to vascular spaces, respectively. Q’ and (Q-L)’ represent biodistribution rate constants in and out of tissue interstitial spaces derived under a quasi-equilibrium assumption.

**Fig C. Comparison between the original and simplified two-pore theory model-based characterization of biodistribution of different molecular sizes of antibody fragments in mice.** Overlay of experimental and model simulations of concentration-time profiles of (A) mAb (150 kDa) and (B) domain antibody (dAb2, 25.6 kDa) in plasma, bone marrow, and spleen in mice after a single intravenous administration of mAb at 3.8 µg or dAb2 at 10 mg/kg in mice. In each panel, symbols represent experimental data. Solid and dashed lines represent model predictions by the simplified and original model [2], respectively, under the lymphatic flow rate reported by Sepp et al [3]. The experimental data were from Shah et al [4] and Sepp et al [3].

**Fig D. Comparison between the original and simplified T cell PBPK model-based characterization of biodistribution of T cells in mice.** Overlay of experimental and model simulations of concentration-time profiles of T cells in blood, bone marrow, and spleen in mice after a single intravenous administration of [^51^Cr]-labelled T cells at 10 μCi per animal. In each panel, symbols represent experimental data. Solid and dashed lines represent model predictions by the simplified and original model [5], respectively. The experimental data were from Khot et al [5].

**Fig E. Comparison between the translated platform model and a model without T cell dynamics on model-simulated biodistribution of AMG211 in patients.** Overlay of experimental positron emission tomography imaging data and model simulation of concentration-time profiles of AMG211 (a carcinoembryonic antigen-targeting TCE, BiTE format, 54 kDa) in blood, bone marrow, and spleen in patients after intravenous infusion of [^89^Zr]AMG211 at 37 MBq/200 μg with cold AMG211 at 1800 μg for 3 hours. Symbols are observed mean for blood and median for tissues (n=4). Solid and dashed lines represent model predictions by the platform model and a mode without T cell dynamics, respectively. The experimental data were from Moek et al [17].

**Fig F. Platform TCE model simulation of concentration-time profiles of free BCMAs, CD3s, and shed BCMAs in blood, bone marrow, lymph node, spleen, and other tissue compartment without a TCE treatment in multiple myeloma patients.**

**Fig G. Platform TCE model characterization of plasma pharmacokinetics of AMG420 in multiple myeloma patients.** Overlay of experimental observations and model simulation of plasma concentration-time profile of AMG420 under continuous intravenous infusion of AMG420 (BiTE format, 54 kDa) at 400 µg/day for 28 days in patients. Symbols and a line represent individual observed data and model prediction, respectively. The experimental data were from Topp et al [18].

**Fig H. Platform TCE model simulation of concentration-time profiles of various species in blood after continuous intravenous administration of AMG420 (a BiTE format, 54 kDa) at 0, 0.01, 0.1, 1, 10, 100, and 1000 mg/kg/week for 4 weeks in patients.** Simulation results were plotted for free TCEs, TAA-TCE-CD3 trimers (immune synapses), TAA-TCE dimers, free CD3s, TCE-CD3 dimers, free TAAs, free shed targets, shed target-TCE dimers, and shed target-TCE-CD3 trimers in blood.

**Fig I. Local parameter sensitivity analysis of the platform model for PF-06863135 (an IgG format, 150 kDa) in patients.** The integrated sensitivity indices of each parameter on immune synapse levels in blood, bone marrow, and lymph node were calculated after subcutaneous injection of PF-06863135 at a 0.5 mg/kg once weekly dose schedule in multiple myeloma patients. The positive sensitivity indices indicate that increase in a parameter value leads to an increase in immune synapse levels.

**Fig J. Local parameter sensitivity analysis of the platform model for AMG420 (a BiTE format, 54 kDa) in patients.** The integrated sensitivity indices of each parameter on immune synapse levels in blood, bone marrow, and lymph node were calculated after intravenous administration of AMG420 at 0.4 mg/day as a continuous intravenous infusion for 28 days in multiple myeloma patients. The positive sensitivity indices indicate that increase in a parameter value leads to an increase in immune synapse levels.

**Fig K. Platform TCE model simulation of two-dimensional contour plots for the effects on immune synapse (TAA-TCE-CD3 trimer) formation in blood, bone marrow, lymph node, spleen, and other tissue compartments by changing expression level of TAAs on tumor cells and CD3s on T cells after subcutaneous TCE (an IgG format, 150 kDa) administration once weekly at 1 mg/kg in patients.** Other parameters were the same as those for PF-06863135.

**Fig L. Platform TCE model simulation of two-dimensional contour plots for the effects on immune synapse (TAA-TCE-CD3 trimer) formation in blood, bone marrow, lymph node, spleen, and other tissue compartments by changing TCE’s equilibrium dissociation constants (kd) for shed targets and expression level of shed targets after subcutaneous TCE (IgG format, 150 kDa) administration once weekly at 1 mg/kg in patients.** Other parameters were the same as those for PF-06863135.

**Fig M. One-compartment TCE model simulation of two-dimensional contour plots for the effects on immune synapse (TAA-TCE-CD3 trimer) formation in blood by changing TCEs’ equilibrium dissociation constants (kd) for TAAs and CD3s after TCE (an IgG format, 150 kDa) administration once weekly at 1 mg/kg in patients.** Other parameters were the same as those for PF-06863135.

**S1 Code SBML code**
